# Preparation and validation of the content of an instrument to assess the quality of services of reception with risk classification in obstetrics

**DOI:** 10.1371/journal.pone.0315816

**Published:** 2024-12-30

**Authors:** Dannielly Azevedo de Oliveira, Douglissandra de Morais Ferreira, Lilian Lira Lisboa, Thaiza Teixeira Xavier Nobre

**Affiliations:** 1 Postgraduate Program in Public Health, Federal University of Rio Grande do Norte, Natal, Rio Grande do Norte, Brazil; 2 Department of Letters, Federal University of Rio Grande do Norte, Natal, Rio Grande do Norte, Brazil; 3 Department of Physiotherapy, Federal University of Rio Grande do Norte, Natal, Rio Grande do Norte, Brazil; Hamadan University of Medical Sciences, ISLAMIC REPUBLIC OF IRAN

## Abstract

**Introduction:**

The expectations and needs of users and those assisting determine the quality of services. It becomes a priority to understand how all elements involved in the care process perceive the quality of the services offered, aiming to intervene promptly and organize them to satisfy the needs of both and improve the assistance provided.

**Objective:**

To develop and carry out content validation of an instrument for evaluating the quality of the Reception service with Risk Classification in obstetrics with evaluation dimensions for users, health professionals, and managers.

**Methodology:**

This is a methodological study for constructing and validating content. Consider the Pasquali Model, following the theoretical pole’s component steps and the empirical’s beginning. An instrument was created and divided into 3 modules: with a dimension for the user (module 1), health workers (module 2), and manager (module 3), created based on the theoretical framework according to Donabedian and the objectives proposed by the World Health Organization and Brazilian Ministry of Health. Validated for content by a committee of judges (experts) specializing in the area of the instrument. The analysis was carried out based on the committee’s agreement rate, Content Validity Index (CVI), and Kappa index for the evaluation and achievement of the ideal parameters of agreement and content validity of the tool among the judges.

**Results:**

The results suggest that the tool has good content validity. CVI, Kappa, and CI were evaluated above 0.8. Item values considered lower than 0.8 were excluded.

**Conclusion:**

The results suggest that the tool has good content validity, and the pilot test stage can be continued to complete the validation process.

## Introduction

Studies evaluating the quality of health services in Brazil have been the object of study over the years thanks to the high values observed in the assistance that must be provided in health care, as well as the increased level of complexity in health (which requires technologies and increasingly sophisticated tools). However, it does not replace medical care [1].

However, when paying attention to the process of evaluating the quality of health services, it is important to bear in mind that this involves both those who use the services and those who produce them. Although the user and provider occupy different positions and views in this process, they both contribute to the development of services [2].

Given this understanding, the expectations and needs of users and those assisting determine the quality of services [2].

Therefore, it becomes a priority to understand how these elements perceive the quality of the services being offered, aiming to identify and analyze the different perceptions to intervene promptly and seek ways to organize them to satisfy the needs of both and promote improvement continuous [1].

Specifically concerning maternal and child health services, to reduce maternal and neonatal morbidity and mortality, pregnant women and newborns need qualified assistance in the labor and birth process through evidence-based practices developed in an environment where they can feel welcomed [3].

To this end, countries provide policies, programs, and agreements that promote improved care. In the reality of Brazil, the Ministry of Health created the Maternal and Child Care Network (Rami) which seeks to restructure the care network for pregnant women and babies throughout the country. In addition to ensuring women have the right to family planning and humanized care during pregnancy, childbirth, and the postpartum period [4].

Within this restructuring process, the need to organize the entrance doors to obstetric care services was observed, in an organized and resolute manner, which has been achieved through the implementation of the Reception with Obstetric Risk Classification (ACCRO) [5, 6], o which constitutes a technical-assistance device that seeks to guarantee access, humanized and timely care to pregnant women seeking obstetric emergencies [7].

Considering what has been presented, good quality of care requires the appropriate use of effective clinical and non-clinical interventions, a reinforced health infrastructure, and positive skills and attitudes on the part of those providing health care, consequently generating better health outcomes and a satisfactory experience for women and service providers [3].

Therefore, when talking about the quality of care during childbirth in health services, it must be taken into account that this will reflect conditions related to physical infrastructure, supplies and inputs, management and human resources available with knowledge, the skills, and capacity to deal with the circumstances of pregnancy and the occurrence of childbirth, which involve normal physiological, social and cultural processes, which may, however, be prone to complications that require immediate interventions to reverse a complication and prevent deaths [3, 8].

Several models guide quality assessment processes in health services [9–14]. Based on these models and the World Health Organization (WHO) health systems approach, a quality of care framework was designed, identifying domains and standards of care that should be targeted to evaluate, improve, and monitor care in health facilities in the context of the health system [3, 8].

In the reality of health studies, it is currently possible to observe an increasing number of measurement instruments to evaluate different outcomes that collaborate with clinical practice, for use in research, as well as in the health assessment of a given population. However, even with the creation of new instruments, many have not been validated or are not validated adequately [15, 16].

The instrument being validated in this study is incorporated into the evaluation of assistance actions aimed at users who are received in the ACCRO sector. The instrument aims to improve the quality of care as it provides the service with a working tool capable of providing data that will provide a care profile of the service that presents the real conditions related to the functioning of the sector.

Considering the need for validated tools that can assess the quality of this type of service, paying attention to the perspective of all individuals involved in the care process, the study presented here seeks to develop and carry out content validation of an instrument for assessing the quality of care. Reception service with Risk Classification in Obstetrics (ACCRO) with assessment dimensions for users (module 1), health professionals (module 2), and managers (module 3).

The current study will use the theoretical model for developing instruments, proposed by Pasquali [17], to verify subjective phenomena, due to the relevance that has been given to psychometric studies. The instrumental development model is based on three major poles, which can be called theoretical, empirical (or experimental), and analytical (or statistical) procedures.

## Materials and methods

This is a methodological study for the construction and validation of instrument content. Considers the proposal of the Pasquali Model [17], following the steps comprising the theoretical pole and the beginning of the empirical pole.

### a. Phase 1—Theory

Initially, the theoretical foundation was carried out on the construct for which it is desired to develop a measuring instrument, that is, the meaning of its properties, the attribution of the dimensionality of these attributes, as well as their constitutive and operational definition.

The development of ACCRO service quality assessment tool in its three modules was developed based on an extensive scoping review that sought to map the assessment of the quality of ACCRO services in the literature, as well as find assessment instruments to be applied in this service already validated.

A broad search strategy was developed and applied to several databases, as well as gray literature in search of articles and primary materials, following a protocol designed for this purpose [18]. Therefore, as no validated tool was found that could evaluate ACCRO services from the perspective of the actors involved in this type of assistance, the research process continued.

### b. Phase 2 –Instrument construction

The constituent items of the instrument were created based on the theoretical framework of the Health Services Quality Assessment proposed by Donabedian [9], considering the structure-process-result assessment, the domains and quality standards of maternal and child care recommended by the WHO [3]^,^ and the guidelines for assistance in ACCRO services directed by the Brazilian Ministry of Health (MS) [5, 6].

Likewise, the items, in their constitution, respected the dimensionality procedures, their conceptualization, and the criteria for their construction, by what Pasquali recommends [17]. Finally, the modules were defined, as presenting 64 items for Module 1 (users; S2 Appendix); 71 items for Module 2 (care professionals; S2 Appendix), and 66 items for Module 3 (managers; S2 Appendix), having been validated for content by a committee of judges (experts) specialized in the area of the instrument.

Eleven people were selected as experts, according to instructions given by Pasquali [17]. In the selection of judges, the following inclusion criteria were considered: Health teachers and/or health professionals with a minimum master’s degree and mastery and/or experience with ACCRO (at least 1 year of experience).

Data collection took place from August 11th to September 31st, 2023 in the virtual environment. A first contact was made via email to present the objectives related to the study, as well as sending an access link to the Free and Informed Consent Form (FICF) written in a Google Forms^®^ type form. Then, after accepting to participate in the study, a new email was sent along with the FICF containing the participant’s acceptance and the researcher’s signature (in a written file in PDF format), in addition to instructions for completing it, along with the instrument assessment, which was composed of the instrument’s domains and questions and the respective judgment criteria [16, 19]. Twenty days were given for the judges to forward the evaluated instrument with the necessary notes. The research only began after authorization from the Research Ethics Committee of the Faculty of Health Sciences of Trairi (FACISA), of the Federal University of Rio Grande do Norte, under opinion number 6.121.102.

Furthermore, for judging the items of an instrument there are twelve criteria, related to Pasquali’s methodological framework, which provide support for validating the content of this instrument. To evaluate each item, the group of judges observed nine of the twelve criteria: behavioral, objectivity, simplicity, clarity, relevance, precision, variety, modality, and credibility [17, 19].

At this stage, the judges also evaluated each item regarding the dimensions of quality according to Donabedian: Structure, Process, and Result. In addition, suggestions were made regarding the inclusion, removal, or modification of items by the judges.

Before the group of judges could evaluate the instrument, there was a semantic evaluation by a professional with a postgraduate degree in Literature to evaluate the understanding of the items for all members of the population to be studied, in a way that can achieve the lowest stratum of the population, as well as the most sophisticated. Thus, this professional transcribed his understanding item by item and made suggestions for other ways of preparing certain items that could be better understood.

After this phase, the instrument was analyzed by the judges regarding the content of each item. The answers provided, following specific guidance directed to the committee, allowed content analysis. The evaluation data were arranged in a spreadsheet created in the Microsoft Excel^®^ program, and the data was imported into the SPSS^®^ statistical software, temporary version 25.0.

At this stage, the initial empirical procedures proposed by Pasquali were carried out [17], to evaluate the psychometric properties of the instrument. To evaluate the content validity of the items, the Content Validity Index (CVI) proposed by Waltz, sStrickland, and Lenz was used [20], who states that the main focus of content validation is to determine whether the items specified in the instrument represent the adequacy the content of the criteria expressed in the instrument.

Taking into account the function of how the instrument was developed, the results obtained were analyzed according to the Content Validation Index and the aforementioned criteria. Likewise, the committee’s agreement index (CI) and Kappa index were observed to evaluate and achieve the ideal parameters of agreement and content validity of the instrument among the judges [19, 21].

## Results

### Expert group profile

All specialists are women, nurses with experience in urgent and emergency obstetric services and ACCRO, mostly with between 6 and 10 years of experience (45.45%). They are aged between 30 and 35 years old (27.27%) and between 41 and 45 years old (27.27%), and are, as a rule, masters (54.55%), working both in assistance (54.55%), as in teaching (54.55%).

They work in the area of Obstetric Nursing (54.55%), having experience in the service in which they assist between 06 and 10 years (54.55%), and for the same period, they work in the current position of which they are part (36.36%). Regarding the time working in maternal and child services, most have worked for more than 15 years (45.45%) in this care context.

The data presented can be observed in Table 1, which provides information regarding the sociodemographic profile of the participants in the group of judges.

**Table 1:** Sociodemographic profile of experts participating in the group of judges.

Profile of experts		Absolute frequency	%
**Age**	30–35 years	3	27.27
	36–40 years	2	18.18
	41–45 years	3	27.27
	45–50 years	1	9.09
	Over 50 years old	2	18.18
**Titration**	Master’s degree	6	54.55
	Doctorate	5	45.45
**Occupation area**	Assistance	6	54.55
	Teaching	6	54.55
	Search	2	18.18
**Current position**	Obstetric nurse	6	54.55
	Teacher	5	45.45
	6–10 years	6	54.55
	11–15 years	1	9.09
	15 years	3	27.27
	Over 15 years old	1	9.09
**Time in current position**	15 years	3	27.27
	6–10 years	4	36.36
	11–15 years	1	9.09
	Over 15 years old	3	27.27
**Time working in maternal and child services**	15 years	2	18.18
	6–10 years	4	36.36
	Over 15 years old	5	45.45
**Length of experience in obstetric urgency and emergency services and reception with obstetric risk classification**	15 years	4	36.36
	6–10 years	5	45.45
	11–15 years	1	9.09
	Over 15 years old	1	9.09
**Total**		**11**	**100.00**

Source: Survey 2023.

### Judges’ analysis

As a result, after content evaluation by the group of judges, the tool was finalized in three modules, module 1 being applied to pregnant and postpartum women who made up the study sample (presenting 52 items to be evaluated), module 2 to be applied to professionals (with 64 items) and 3 to be aimed at managers of such services (with 53 items).

#### 1. Statistical analysis Module 1

About Module 1, developed to evaluate users served by the ACCRO service, considering the statistical rigor necessary to prepare the instrument, the cutoff point for CI, CVI, and Kappa was defined as values equal to or greater than 0.80. Instrument items that obtained values lower than this were excluded from the study. The data can be seen in Table 2.

**Table 2:** Validation of the instrument for assessing the quality of reception services with obstetric risk classification for users. Module 1.

Item	Behavioral			Objectivity			Simplicity			Clarity			Relevance			Precision			Variety			Modality			Credibility		
Q^a^	CI^b^	Kappa^c^	CVI^d^	CI	Kappa	CVI	CI	Kappa	CVI	CI	Kappa	CVI	CI	Kappa	CVI	CI	Kappa	CVI	CI	Kappa	CVI	CI	Kappa	CVI	CI	Kappa	CVI
**Q1**	1.00	1.00	1.00	1.00	1.00	1.00	1.00	1.00	1.00	1.00	1.00	1.00	1.00	1.00	1.00	0.80	0.60	0.90	1.00	1.00	1.00	1.00	1.00	1.00	1.00	1.00	1.00
**Q2**	1.00	1.00	1.00	1.00	1.00	1.00	1.00	1.00	1.00	1.00	1.00	1.00	1.00	1.00	1.00	1.00	1.00	1.00	1.00	1.00	1.00	1.00	1.00	1.00	1.00	1.00	1.00
**Q3**	1.00	1.00	1.00	1.00	1.00	1.00	1.00	1.00	1.00	1.00	1.00	1.00	1.00	1.00	1.00	1.00	1.00	1.00	1.00	1.00	1.00	1.00	1.00	1.00	1.00	1.00	1.00
**Q4**	1.00	1.00	1.00	1.00	1.00	1.00	1.00	1.00	1.00	1.00	1.00	1.00	1.00	1.00	1.00	1.00	1.00	1.00	1.00	1.00	1.00	1.00	1.00	1.00	1.00	1.00	1.00
**Q5**	1.00	1.00	1.00	1.00	1.00	1.00	1.00	1.00	1.00	1.00	1.00	1.00	1.00	1.00	1.00	1.00	1.00	1.00	1.00	1.00	1.00	1.00	1.00	1.00	1.00	1.00	1.00
**Q6**	0.80	0.60	0.90	0.80	0.60	0.90	0.80	0.60	0.90	1.00	1.00	1.00	1.00	1.00	1.00	1.00	1.00	1.00	1.00	1.00	1.00	1.00	1.00	1.00	1.00	1.00	1.00
**Q7**	1.00	1.00	1.00	1.00	1.00	1.00	1.00	1.00	1.00	1.00	1.00	1.00	1.00	1.00	1.00	1.00	1.00	1.00	1.00	1.00	1.00	1.00	1.00	1.00	1.00	1.00	1.00
**Q8**	1.00	1.00	1.00	1.00	1.00	1.00	1.00	1.00	1.00	1.00	1.00	1.00	1.00	1.00	1.00	1.00	1.00	1.00	1.00	1.00	1.00	1.00	1.00	1.00	1.00	1.00	1.00
**Q9**	1.00	1.00	1.00	1.00	1.00	1.00	1.00	1.00	1.00	1.00	1.00	1.00	1.00	1.00	1.00	1.00	1.00	1.00	1.00	1.00	1.00	1.00	1.00	1.00	1.00	1.00	1.00
**Q10**	1.00	1.00	1.00	1.00	1.00	1.00	1.00	1.00	1.00	1.00	1.00	1.00	1.00	1.00	1.00	1.00	1.00	1.00	1.00	1.00	1.00	1.00	1.00	1.00	1.00	1.00	1.00
**Q11**	1.00	1.00	1.00	1.00	1.00	1.00	1.00	1.00	1.00	1.00	1.00	1.00	1.00	1.00	1.00	1.00	1.00	1.00	1.00	1.00	1.00	1.00	1.00	1.00	1.00	1.00	1.00
**Q12**	1.00	1.00	1.00	1.00	1.00	1.00	1.00	1.00	1.00	1.00	1.00	1.00	1.00	1.00	1.00	1.00	1.00	1.00	1.00	1.00	1.00	1.00	1.00	1.00	1.00	1.00	1.00
**Q13**	0.80	0.60	0.90	0.80	0.60	0.90	1.00	1.00	1.00	1.00	1.00	1.00	1.00	1.00	1.00	1.00	1.00	1.00	1.00	1.00	1.00	1.00	1.00	1.00	1.00	1.00	1.00
**Q14**	1.00	1.00	1.00	1.00	1.00	1.00	1.00	1.00	1.00	1.00	1.00	1.00	1.00	1.00	1.00	1.00	1.00	1.00	1.00	1.00	1.00	1.00	1.00	1.00	1.00	1.00	1.00
**Q15**	1.00	1.00	1.00	1.00	1.00	1.00	1.00	1.00	1.00	1.00	1.00	1.00	1.00	1.00	1.00	1.00	1.00	1.00	1.00	1.00	1.00	1.00	1.00	1.00	1.00	1.00	1.00
**Q16**	1.00	1.00	1.00	1.00	1.00	1.00	1.00	1.00	1.00	1.00	1.00	1.00	1.00	1.00	1.00	1.00	1.00	1.00	1.00	1.00	1.00	1.00	1.00	1.00	1.00	1.00	1.00
**Q17**	1.00	1.00	1.00	1.00	1.00	1.00	1.00	1.00	1.00	1.00	1.00	1.00	1.00	1.00	1.00	1.00	1.00	1.00	1.00	1.00	1.00	1.00	1.00	1.00	1.00	1.00	1.00
**Q18**	1.00	1.00	1.00	1.00	1.00	1.00	1.00	1.00	1.00	1.00	1.00	1.00	1.00	1.00	1.00	1.00	1.00	1.00	1.00	1.00	1.00	1.00	1.00	1.00	1.00	1.00	1.00
**Q19**	1.00	1.00	1.00	1.00	1.00	1.00	1.00	1.00	1.00	1.00	1.00	1.00	1.00	1.00	1.00	1.00	1.00	1.00	1.00	1.00	1.00	1.00	1.00	1.00	1.00	1.00	1.00
**Q20**	1.00	1.00	1.00	1.00	1.00	1.00	1.00	1.00	1.00	1.00	1.00	1.00	1.00	1.00	1.00	1.00	1.00	1.00	1.00	1.00	1.00	1.00	1.00	1.00	1.00	1.00	1.00
**Q21**	0.64	0.50	0.80	0.64	0.50	0.80	1.00	1.00	1.00	0.80	0.60	0.90	1.00	1.00	1.00	0.80	0.60	0.90	1.00	1.00	1.00	1.00	1.00	1.00	1.00	1.00	1.00
**Q22**	0.78	0.56	0.89	0.78	0.56	0.89	0.78	0.56	0.89	0.78	0.56	0.89	0.78	0.56	0.89	0.78	0.56	0.89	1.00	1.00	1.00	1.00	1.00	1.00	0.78	0.56	0.89
**Q23**	1.00	1.00	1.00	1.00	1.00	1.00	1.00	1.00	1.00	1.00	1.00	1.00	1.00	1.00	1.00	1.00	1.00	1.00	1.00	1.00	1.00	1.00	1.00	1.00	1.00	1.00	1.00
**Q24**	1.00	1.00	1.00	1.00	1.00	1.00	1.00	1.00	1.00	1.00	1.00	1.00	1.00	1.00	1.00	1.00	1.00	1.00	1.00	1.00	1.00	1.00	1.00	1.00	1.00	1.00	1.00
**Q25**	1.00	1.00	1.00	1.00	1.00	1.00	1.00	1.00	1.00	1.00	1.00	1.00	1.00	1.00	1.00	1.00	1.00	1.00	1.00	1.00	1.00	1.00	1.00	1.00	1.00	1.00	1.00
**Q26**	1.00	1.00	1.00	1.00	1.00	1.00	1.00	1.00	1.00	1.00	1.00	1.00	1.00	1.00	1.00	1.00	1.00	1.00	1.00	1.00	1.00	1.00	1.00	1.00	1.00	1.00	1.00
**Q27**	1.00	1.00	1.00	1.00	1.00	1.00	1.00	1.00	1.00	1.00	1.00	1.00	1.00	1.00	1.00	1.00	1.00	1.00	1.00	1.00	1.00	1.00	1.00	1.00	1.00	1.00	1.00
**Q28**	1.00	1.00	1.00	1.00	1.00	1.00	1.00	1.00	1.00	1.00	1.00	1.00	1.00	1.00	1.00	1.00	1.00	1.00	1.00	1.00	1.00	1.00	1.00	1.00	1.00	1.00	1.00
**Q29**	1.00	1.00	1.00	1.00	1.00	1.00	1.00	1.00	1.00	1.00	1.00	1.00	1.00	1.00	1.00	1.00	1.00	1.00	1.00	1.00	1.00	1.00	1.00	1.00	1.00	1.00	1.00
**Q30**	0.80	0.60	0.90	0.80	0.60	0.90	0.80	0.60	0.90	0.80	0.60	0.90	1.00	1.00	1.00	0.80	0.60	0.90	1.00	1.00	1.00	1.00	1.00	1.00	0.80	0.60	0.90
**Q31**	1.00	1.00	1.00	1.00	1.00	1.00	1.00	1.00	1.00	1.00	1.00	1.00	1.00	1.00	1.00	0.80	0.60	0.90	0.80	0.60	0.90	0.80	0.60	0.90	0.80	0.60	0.90
**Q32**	1.00	1.00	1.00	1.00	1.00	1.00	1.00	1.00	1.00	1.00	1.00	1.00	1.00	1.00	1.00	1.00	1.00	1.00	1.00	1.00	1.00	1.00	1.00	1.00	1.00	1.00	1.00
**Q33**	1.00	1.00	1.00	1.00	1.00	1.00	1.00	1.00	1.00	1.00	1.00	1.00	1.00	1.00	1.00	1.00	1.00	1.00	1.00	1.00	1.00	1.00	1.00	1.00	1.00	1.00	1.00
**Q34**	0.80	0.60	0.90	0.80	0.60	0.90	0.80	0.60	0.90	0.80	0.60	0.90	0.80	0.60	0.90	0.80	0.60	0.90	1.00	1.00	1.00	1.00	1.00	1.00	1.00	1.00	1.00
**Q35**	1.00	1.00	1.00	1.00	1.00	1.00	1.00	1.00	1.00	1.00	1.00	1.00	1.00	1.00	1.00	1.00	1.00	1.00	1.00	1.00	1.00	1.00	1.00	1.00	1.00	1.00	1.00
**Q36**	1.00	1.00	1.00	1.00	1.00	1.00	1.00	1.00	1.00	1.00	1.00	1.00	1.00	1.00	1.00	1.00	1.00	1.00	1.00	1.00	1.00	1.00	1.00	1.00	1.00	1.00	1.00
**Q37**	1.00	1.00	1.00	1.00	1.00	1.00	1.00	1.00	1.00	1.00	1.00	1.00	1.00	1.00	1.00	0.78	0.56	0.89	1.00	1.00	1.00	1.00	1.00	1.00	0.78	0.56	0.89
**Q38**	1.00	1.00	1.00	1.00	1.00	1.00	1.00	1.00	1.00	1.00	1.00	1.00	1.00	1.00	1.00	1.00	1.00	1.00	1.00	1.00	1.00	1.00	1.00	1.00	1.00	1.00	1.00
**Q39**	1.00	1.00	1.00	1.00	1.00	1.00	1.00	1.00	1.00	1.00	1.00	1.00	1.00	1.00	1.00	1.00	1.00	1.00	1.00	1.00	1.00	1.00	1.00	1.00	1.00	1.00	1.00
**Q40**	1.00	1.00	1.00	1.00	1.00	1.00	1.00	1.00	1.00	1.00	1.00	1.00	1.00	1.00	1.00	1.00	1.00	1.00	1.00	1.00	1.00	1.00	1.00	1.00	1.00	1.00	1.00
**Q41**	1.00	1.00	1.00	1.00	1.00	1.00	1.00	1.00	1.00	1.00	1.00	1.00	1.00	1.00	1.00	1.00	1.00	1.00	1.00	1.00	1.00	1.00	1.00	1.00	1.00	1.00	1.00
**Q42**	1.00	1.00	1.00	1.00	1.00	1.00	1.00	1.00	1.00	1.00	1.00	1.00	1.00	1.00	1.00	1.00	1.00	1.00	1.00	1.00	1.00	1.00	1.00	1.00	1.00	1.00	1.00
**Q43**	1.00	1.00	1.00	1.00	1.00	1.00	1.00	1.00	1.00	1.00	1.00	1.00	1.00	1.00	1.00	1.00	1.00	1.00	1.00	1.00	1.00	1.00	1.00	1.00	1.00	1.00	1.00
**Q44**	1.00	1.00	1.00	1.00	1.00	1.00	1.00	1.00	1.00	1.00	1.00	1.00	1.00	1.00	1.00	1.00	1.00	1.00	1.00	1.00	1.00	1.00	1.00	1.00	1.00	1.00	1.00
**Q45**	1.00	1.00	1.00	1.00	1.00	1.00	1.00	1.00	1.00	1.00	1.00	1.00	1.00	1.00	1.00	1.00	1.00	1.00	1.00	1.00	1.00	1.00	1.00	1.00	1.00	1.00	1.00
**Q46**	1.00	1.00	1.00	1.00	1.00	1.00	1.00	1.00	1.00	1.00	1.00	1.00	1.00	1.00	1.00	1.00	1.00	1.00	1.00	1.00	1.00	1.00	1.00	1.00	1.00	1.00	1.00
**Q47**	1.00	1.00	1.00	1.00	1.00	1.00	1.00	1.00	1.00	1.00	1.00	1.00	1.00	1.00	1.00	1.00	1.00	1.00	1.00	1.00	1.00	1.00	1.00	1.00	1.00	1.00	1.00
**Q48**	1.00	1.00	1.00	1.00	1.00	1.00	1.00	1.00	1.00	1.00	1.00	1.00	1.00	1.00	1.00	1.00	1.00	1.00	1.00	1.00	1.00	1.00	1.00	1.00	1.00	1.00	1.00
**Q49**	1.00	1.00	1.00	1.00	1.00	1.00	1.00	1.00	1.00	1.00	1.00	1.00	1.00	1.00	1.00	1.00	1.00	1.00	1.00	1.00	1.00	1.00	1.00	1.00	1.00	1.00	1.00
**Q50**	1.00	1.00	1.00	1.00	1.00	1.00	1.00	1.00	1.00	1.00	1.00	1.00	1.00	1.00	1.00	0.80	0.60	0.90	1.00	1.00	1.00	1.00	1.00	1.00	0.80	0.60	0.90
**Q51**	1.00	1.00	1.00	1.00	1.00	1.00	1.00	1.00	1.00	1.00	1.00	1.00	1.00	1.00	1.00	1.00	1.00	1.00	1.00	1.00	1.00	1.00	1.00	1.00	1.00	1.00	1.00
**Q52**	1.00	1.00	1.00	1.00	1.00	1.00	1.00	1.00	1.00	1.00	1.00	1.00	1.00	1.00	1.00	1.00	1.00	1.00	1.00	1.00	1.00	1.00	1.00	1.00	1.00	1.00	1.00
**Q53**	1.00	1.00	1.00	1.00	1.00	1.00	1.00	1.00	1.00	1.00	1.00	1.00	1.00	1.00	1.00	1.00	1.00	1.00	1.00	1.00	1.00	1.00	1.00	1.00	1.00	1.00	1.00
**Q54**	1.00	1.00	1.00	1.00	1.00	1.00	1.00	1.00	1.00	1.00	1.00	1.00	1.00	1.00	1.00	1.00	1.00	1.00	1.00	1.00	1.00	1.00	1.00	1.00	1.00	1.00	1.00
**Q55**	1.00	1.00	1.00	1.00	1.00	1.00	1.00	1.00	1.00	1.00	1.00	1.00	1.00	1.00	1.00	0.80	0.60	0.90	1.00	1.00	1.00	1.00	1.00	1.00	0.80	0.60	0.90
**Q56**	1.00	1.00	1.00	1.00	1.00	1.00	1.00	1.00	1.00	1.00	1.00	1.00	1.00	1.00	1.00	1.00	1.00	1.00	1.00	1.00	1.00	1.00	1.00	1.00	1.00	1.00	1.00
**Q57**	1.00	1.00	1.00	1.00	1.00	1.00	1.00	1.00	1.00	1.00	1.00	1.00	1.00	1.00	1.00	0.80	0.60	0.90	1.00	1.00	1.00	1.00	1.00	1.00	0.80	0.60	0.90
**Q58**	1.00	1.00	1.00	1.00	1.00	1.00	1.00	1.00	1.00	1.00	1.00	1.00	1.00	1.00	1.00	1.00	1.00	1.00	1.00	1.00	1.00	1.00	1.00	1.00	1.00	1.00	1.00
**Q59**	1.00	1.00	1.00	1.00	1.00	1.00	1.00	1.00	1.00	1.00	1.00	1.00	1.00	1.00	1.00	1.00	1.00	1.00	1.00	1.00	1.00	1.00	1.00	1.00	1.00	1.00	1.00
**Q60**	1.00	1.00	1.00	1.00	1.00	1.00	1.00	1.00	1.00	1.00	1.00	1.00	1.00	1.00	1.00	1.00	1.00	1.00	1.00	1.00	1.00	1.00	1.00	1.00	1.00	1.00	1.00
**Q61**	1.00	1.00	1.00	1.00	1.00	1.00	1.00	1.00	1.00	1.00	1.00	1.00	1.00	1.00	1.00	1.00	1.00	1.00	1.00	1.00	1.00	1.00	1.00	1.00	1.00	1.00	1.00
**Q62**	1.00	1.00	1.00	1.00	1.00	1.00	1.00	1.00	1.00	1.00	1.00	1.00	1.00	1.00	1.00	1.00	1.00	1.00	1.00	1.00	1.00	1.00	1.00	1.00	1.00	1.00	1.00
**Q63**	1.00	1.00	1.00	1.00	1.00	1.00	1.00	1.00	1.00	1.00	1.00	1.00	1.00	1.00	1.00	1.00	1.00	1.00	1.00	1.00	1.00	1.00	1.00	1.00	1.00	1.00	1.00
**Q64**	1.00	1.00	1.00	1.00	1.00	1.00	1.00	1.00	1.00	1.00	1.00	1.00	1.00	1.00	1.00	1.00	1.00	1.00	1.00	1.00	1.00	1.00	1.00	1.00	1.00	1.00	1.00

Source: Survey 2023.

^a^Q = instrument item

^b^CI = Committee agreement index

^c^kappa = Kappa index

^d^CVI = Content Validity Index.

Specifically presenting the agreement index between experts (IC), we have the following items below 0.80: Behavioral (21 and 22); Objectivity (21 and 22); Simplicity (22); Clarity (22); Relevance (22); Accuracy (22 and 37) and Credibility (22 and 37).

Using the Kappa index, which assesses the degree of agreement between evaluators on the items under study, we have the following items below 0.80: Behavioral (6, 13, 21, 22, 30 and 34); Objectivity (6, 13, 21, 22, 30 and 34); Simplicity (6, 22, 30 and 34); Clarity (21, 22, 30 and 34); Relevance (22 and 34); Accuracy (1, 21, 22, 30, 31, 34, 37, 50, 55 and 57); Variety (31); Modality (31) and Credibility (22, 30, 31, 37, 50, 55 and 57).

Furthermore, through the CVI, defining a cutoff point of 0.80, all items under analysis are classified as adequate. Therefore, the instrument is being validated with experts, as can be seen in Table 2.

#### 2. Statistical analysis Module 2

About Module 2, developed for the evaluation of professionals who assist in the ACCRO service, items that were contained in Module 1 and that had already been evaluated by the group were excluded from the evaluation, so that the process would not be repeated. Thus, five items, previously evaluated, were not presented in the judgment of this module.

Considering the statistical rigor necessary to develop the instrument, the cutoff point for CI, CVI, and Kappa was defined as values equal to or greater than 0.80. Instrument items that obtained values lower than this were excluded from the study. The data can be seen in Table 3.

**Table 3:** Validation of the instrument for assessing the quality of reception services with obstetric risk classification for professionals. Module 2.

Item	Behavioral			Objectivity			Simplicity			Clarity			Relevance			Precision			Variety			Modality			Credibility		
Q^a^	CI^b^	Kappa^c^	CVI^d^	CI	Kappa	CVI	CI	Kappa	CVI	CI	Kappa	CVI	CI	Kappa	CVI	CI	Kappa	CVI	CI	Kappa	CVI	CI	Kappa	CVI	CI	Kappa	CVI
**Q1**	1.00	1.00	1.00	1.00	1.00	1.00	1.00	1.00	1.00	1.00	1.00	1.00	1.00	1.00	1.00	1.00	1.00	1.00	1.00	1.00	1.00	1.00	1.00	1.00	1.00	1.00	1.00
**Q2**	1.00	1.00	1.00	1.00	1.00	1.00	1.00	1.00	1.00	1.00	1.00	1.00	1.00	1.00	1.00	1.00	1.00	1.00	1.00	1.00	1.00	1.00	1.00	1.00	1.00	1.00	1.00
**Q3**	1.00	1.00	1.00	1.00	1.00	1.00	1.00	1.00	1.00	1.00	1.00	1.00	1.00	1.00	1.00	1.00	1.00	1.00	1.00	1.00	1.00	1.00	1.00	1.00	1.00	1.00	1.00
**Q4**	1.00	1.00	1.00	1.00	1.00	1.00	1.00	1.00	1.00	1.00	1.00	1.00	1.00	1.00	1.00	1.00	1.00	1.00	1.00	1.00	1.00	1.00	1.00	1.00	1.00	1.00	1.00
**Q5**	1.00	1.00	1.00	1.00	1.00	1.00	1.00	1.00	1.00	1.00	1.00	1.00	1.00	1.00	1.00	1.00	1.00	1.00	0.82	0.64	0.91	1.00	1.00	1.00	1.00	1.00	1.00
**Q6**	1.00	1.00	1.00	1.00	1.00	1.00	1.00	1.00	1.00	1.00	1.00	1.00	1.00	1.00	1.00	1.00	1.00	1.00	1.00	1.00	1.00	1.00	1.00	1.00	1.00	1.00	1.00
**Q7**	1.00	1.00	1.00	1.00	1.00	1.00	1.00	1.00	1.00	1.00	1.00	1.00	1.00	1.00	1.00	1.00	1.00	1.00	1.00	1.00	1.00	1.00	1.00	1.00	1.00	1.00	1.00
**Q8**	0.82	0.64	0.91	0.82	0.64	0.91	0.82	0.64	0.91	0.82	0.64	0.91	1.00	1.00	1.00	0.82	0.64	0.91	1.00	1.00	1.00	1.00	1.00	1.00	0.82	0.64	0.91
**Q9**	1.00	1.00	1.00	1.00	1.00	1.00	1.00	1.00	1.00	1.00	1.00	1.00	1.00	1.00	1.00	1.00	1.00	1.00	1.00	1.00	1.00	1.00	1.00	1.00	1.00	1.00	1.00
**Q10**	1.00	1.00	1.00	1.00	1.00	1.00	1.00	1.00	1.00	1.00	1.00	1.00	1.00	1.00	1.00	1.00	1.00	1.00	1.00	1.00	1.00	1.00	1.00	1.00	1.00	1.00	1.00
**Q11**	1.00	1.00	1.00	1.00	1.00	1.00	1.00	1.00	1.00	1.00	1.00	1.00	1.00	1.00	1.00	1.00	1.00	1.00	1.00	1.00	1.00	1.00	1.00	1.00	1.00	1.00	1.00
**Q12**	1.00	1.00	1.00	1.00	1.00	1.00	1.00	1.00	1.00	1.00	1.00	1.00	1.00	1.00	1.00	1.00	1.00	1.00	1.00	1.00	1.00	1.00	1.00	1.00	1.00	1.00	1.00
**Q13**	1.00	1.00	1.00	1.00	1.00	1.00	1.00	1.00	1.00	1.00	1.00	1.00	1.00	1.00	1.00	1.00	1.00	1.00	1.00	1.00	1.00	1.00	1.00	1.00	1.00	1.00	1.00
**Q14**	1.00	1.00	1.00	1.00	1.00	1.00	1.00	1.00	1.00	1.00	1.00	1.00	1.00	1.00	1.00	1.00	1.00	1.00	1.00	1.00	1.00	1.00	1.00	1.00	1.00	1.00	1.00
**Q15**	1.00	1.00	1.00	1.00	1.00	1.00	1.00	1.00	1.00	1.00	1.00	1.00	1.00	1.00	1.00	1.00	1.00	1.00	1.00	1.00	1.00	1.00	1.00	1.00	1.00	1.00	1.00
**Q16**	1.00	1.00	1.00	1.00	1.00	1.00	1.00	1.00	1.00	1.00	1.00	1.00	1.00	1.00	1.00	1.00	1.00	1.00	1.00	1.00	1.00	1.00	1.00	1.00	1.00	1.00	1.00
**Q17**	1.00	1.00	1.00	1.00	1.00	1.00	1.00	1.00	1.00	1.00	1.00	1.00	1.00	1.00	1.00	1.00	1.00	1.00	1.00	1.00	1.00	1.00	1.00	1.00	1.00	1.00	1.00
**Q18**	1.00	1.00	1.00	1.00	1.00	1.00	1.00	1.00	1.00	1.00	1.00	1.00	1.00	1.00	1.00	1.00	1.00	1.00	1.00	1.00	1.00	1.00	1.00	1.00	1.00	1.00	1.00
**Q19**	1.00	1.00	1.00	1.00	1.00	1.00	1.00	1.00	1.00	1.00	1.00	1.00	1.00	1.00	1.00	1.00	1.00	1.00	1.00	1.00	1.00	1.00	1.00	1.00	1.00	1.00	1.00
**Q20**	1.00	1.00	1.00	1.00	1.00	1.00	1.00	1.00	1.00	1.00	1.00	1.00	1.00	1.00	1.00	1.00	1.00	1.00	1.00	1.00	1.00	1.00	1.00	1.00	1.00	1.00	1.00
**Q22**	1.00	1.00	1.00	1.00	1.00	1.00	1.00	1.00	1.00	1.00	1.00	1.00	1.00	1.00	1.00	1.00	1.00	1.00	1.00	1.00	1.00	1.00	1.00	1.00	1.00	1.00	1.00
**Q23**	1.00	1.00	1.00	1.00	1.00	1.00	1.00	1.00	1.00	1.00	1.00	1.00	1.00	1.00	1.00	1.00	1.00	1.00	1.00	1.00	1.00	1.00	1.00	1.00	1.00	1.00	1.00
**Q24**	1.00	1.00	1.00	1.00	1.00	1.00	1.00	1.00	1.00	1.00	1.00	1.00	1.00	1.00	1.00	1.00	1.00	1.00	1.00	1.00	1.00	1.00	1.00	1.00	1.00	1.00	1.00
**Q25**	1.00	1.00	1.00	1.00	1.00	1.00	1.00	1.00	1.00	1.00	1.00	1.00	1.00	1.00	1.00	1.00	1.00	1.00	1.00	1.00	1.00	1.00	1.00	1.00	1.00	1.00	1.00
**Q26**	1.00	1.00	1.00	1.00	1.00	1.00	1.00	1.00	1.00	1.00	1.00	1.00	1.00	1.00	1.00	1.00	1.00	1.00	1.00	1.00	1.00	1.00	1.00	1.00	1.00	1.00	1.00
**Q27**	1.00	1.00	1.00	1.00	1.00	1.00	1.00	1.00	1.00	1.00	1.00	1.00	1.00	1.00	1.00	1.00	1.00	1.00	1.00	1.00	1.00	1.00	1.00	1.00	1.00	1.00	1.00
**Q28**	1.00	1.00	1.00	1.00	1.00	1.00	1.00	1.00	1.00	1.00	1.00	1.00	1.00	1.00	1.00	1.00	1.00	1.00	1.00	1.00	1.00	1.00	1.00	1.00	1.00	1.00	1.00
**Q29**	1.00	1.00	1.00	1.00	1.00	1.00	1.00	1.00	1.00	1.00	1.00	1.00	1.00	1.00	1.00	1.00	1.00	1.00	1.00	1.00	1.00	1.00	1.00	1.00	1.00	1.00	1.00
**Q30**	1.00	1.00	1.00	1.00	1.00	1.00	1.00	1.00	1.00	1.00	1.00	1.00	1.00	1.00	1.00	1.00	1.00	1.00	1.00	1.00	1.00	1.00	1.00	1.00	1.00	1.00	1.00
**Q31**	1.00	1.00	1.00	1.00	1.00	1.00	1.00	1.00	1.00	1.00	1.00	1.00	1.00	1.00	1.00	1.00	1.00	1.00	1.00	1.00	1.00	1.00	1.00	1.00	1.00	1.00	1.00
**Q32**	1.00	1.00	1.00	1.00	1.00	1.00	1.00	1.00	1.00	1.00	1.00	1.00	1.00	1.00	1.00	1.00	1.00	1.00	1.00	1.00	1.00	1.00	1.00	1.00	1.00	1.00	1.00
**Q33**	1.00	1.00	1.00	1.00	1.00	1.00	1.00	1.00	1.00	1.00	1.00	1.00	1.00	1.00	1.00	1.00	1.00	1.00	0.82	0.64	0.91	1.00	1.00	1.00	1.00	1.00	1.00
**Q34**	1.00	1.00	1.00	1.00	1.00	1.00	1.00	1.00	1.00	1.00	1.00	1.00	1.00	1.00	1.00	1.00	1.00	1.00	1.00	1.00	1.00	1.00	1.00	1.00	1.00	1.00	1.00
**Q35**	0.82	0.64	0.91	0.82	0.64	0.91	0.82	0.64	0.91	0.82	0.64	0.91	0.82	0.64	0.91	0.82	0.64	0.91	0.82	0.64	0.91	0.82	0.64	0.91	0.82	0.64	0.91
**Q36**	1.00	1.00	1.00	1.00	1.00	1.00	1.00	1.00	1.00	1.00	1.00	1.00	1.00	1.00	1.00	1.00	1.00	1.00	1.00	1.00	1.00	1.00	1.00	1.00	1.00	1.00	1.00
**Q37**	1.00	1.00	1.00	1.00	1.00	1.00	1.00	1.00	1.00	1.00	1.00	1.00	1.00	1.00	1.00	1.00	1.00	1.00	1.00	1.00	1.00	1.00	1.00	1.00	1.00	1.00	1.00
**Q38**	1.00	1.00	1.00	1.00	1.00	1.00	1.00	1.00	1.00	1.00	1.00	1.00	1.00	1.00	1.00	1.00	1.00	1.00	1.00	1.00	1.00	1.00	1.00	1.00	1.00	1.00	1.00
**Q39**	1.00	1.00	1.00	1.00	1.00	1.00	1.00	1.00	1.00	1.00	1.00	1.00	1.00	1.00	1.00	1.00	1.00	1.00	1.00	1.00	1.00	1.00	1.00	1.00	1.00	1.00	1.00
**Q40**	1.00	1.00	1.00	1.00	1.00	1.00	1.00	1.00	1.00	1.00	1.00	1.00	1.00	1.00	1.00	1.00	1.00	1.00	1.00	1.00	1.00	1.00	1.00	1.00	1.00	1.00	1.00
**Q41**	1.00	1.00	1.00	1.00	1.00	1.00	1.00	1.00	1.00	1.00	1.00	1.00	1.00	1.00	1.00	1.00	1.00	1.00	1.00	1.00	1.00	1.00	1.00	1.00	1.00	1.00	1.00
**Q42**	1.00	1.00	1.00	1.00	1.00	1.00	1.00	1.00	1.00	1.00	1.00	1.00	1.00	1.00	1.00	1.00	1.00	1.00	1.00	1.00	1.00	1.00	1.00	1.00	1.00	1.00	1.00
**Q43**	1.00	1.00	1.00	1.00	1.00	1.00	1.00	1.00	1.00	1.00	1.00	1.00	1.00	1.00	1.00	1.00	1.00	1.00	1.00	1.00	1.00	1.00	1.00	1.00	1.00	1.00	1.00
**Q44**	1.00	1.00	1.00	1.00	1.00	1.00	1.00	1.00	1.00	1.00	1.00	1.00	1.00	1.00	1.00	1.00	1.00	1.00	1.00	1.00	1.00	1.00	1.00	1.00	1.00	1.00	1.00
**Q45**	1.00	1.00	1.00	1.00	1.00	1.00	1.00	1.00	1.00	1.00	1.00	1.00	1.00	1.00	1.00	1.00	1.00	1.00	1.00	1.00	1.00	1.00	1.00	1.00	1.00	1.00	1.00
**Q46**	1.00	1.00	1.00	1.00	1.00	1.00	1.00	1.00	1.00	1.00	1.00	1.00	1.00	1.00	1.00	1.00	1.00	1.00	1.00	1.00	1.00	1.00	1.00	1.00	1.00	1.00	1.00
**Q47**	1.00	1.00	1.00	1.00	1.00	1.00	1.00	1.00	1.00	1.00	1.00	1.00	1.00	1.00	1.00	1.00	1.00	1.00	1.00	1.00	1.00	1.00	1.00	1.00	1.00	1.00	1.00
**Q48**	1.00	1.00	1.00	1.00	1.00	1.00	1.00	1.00	1.00	1.00	1.00	1.00	1.00	1.00	1.00	1.00	1.00	1.00	1.00	1.00	1.00	1.00	1.00	1.00	1.00	1.00	1.00
**Q49**	1.00	1.00	1.00	1.00	1.00	1.00	1.00	1.00	1.00	1.00	1.00	1.00	1.00	1.00	1.00	1.00	1.00	1.00	1.00	1.00	1.00	1.00	1.00	1.00	1.00	1.00	1.00
**Q50**	1.00	1.00	1.00	1.00	1.00	1.00	1.00	1.00	1.00	1.00	1.00	1.00	1.00	1.00	1.00	1.00	1.00	1.00	1.00	1.00	1.00	1.00	1.00	1.00	1.00	1.00	1.00
**Q51**	1.00	1.00	1.00	1.00	1.00	1.00	1.00	1.00	1.00	1.00	1.00	1.00	1.00	1.00	1.00	1.00	1.00	1.00	1.00	1.00	1.00	1.00	1.00	1.00	1.00	1.00	1.00
**Q52**	1.00	1.00	1.00	1.00	1.00	1.00	1.00	1.00	1.00	1.00	1.00	1.00	1.00	1.00	1.00	1.00	1.00	1.00	1.00	1.00	1.00	1.00	1.00	1.00	1.00	1.00	1.00
**Q53**	1.00	1.00	1.00	1.00	1.00	1.00	1.00	1.00	1.00	1.00	1.00	1.00	1.00	1.00	1.00	1.00	1.00	1.00	1.00	1.00	1.00	1.00	1.00	1.00	1.00	1.00	1.00
**Q56**	1.00	1.00	1.00	1.00	1.00	1.00	1.00	1.00	1.00	1.00	1.00	1.00	1.00	1.00	1.00	1.00	1.00	1.00	1.00	1.00	1.00	1.00	1.00	1.00	1.00	1.00	1.00
**Q57**	1.00	1.00	1.00	1.00	1.00	1.00	1.00	1.00	1.00	1.00	1.00	1.00	1.00	1.00	1.00	1.00	1.00	1.00	1.00	1.00	1.00	1.00	1.00	1.00	1.00	1.00	1.00
**Q58**	1.00	1.00	1.00	1.00	1.00	1.00	1.00	1.00	1.00	1.00	1.00	1.00	1.00	1.00	1.00	1.00	1.00	1.00	1.00	1.00	1.00	1.00	1.00	1.00	1.00	1.00	1.00
**Q60**	1.00	1.00	1.00	1.00	1.00	1.00	1.00	1.00	1.00	1.00	1.00	1.00	1.00	1.00	1.00	1.00	1.00	1.00	1.00	1.00	1.00	1.00	1.00	1.00	1.00	1.00	1.00
**Q61**	1.00	1.00	1.00	1.00	1.00	1.00	1.00	1.00	1.00	1.00	1.00	1.00	1.00	1.00	1.00	1.00	1.00	1.00	1.00	1.00	1.00	1.00	1.00	1.00	1.00	1.00	1.00
**Q62**	1.00	1.00	1.00	1.00	1.00	1.00	1.00	1.00	1.00	1.00	1.00	1.00	1.00	1.00	1.00	1.00	1.00	1.00	1.00	1.00	1.00	1.00	1.00	1.00	1.00	1.00	1.00
**Q63**	1.00	1.00	1.00	1.00	1.00	1.00	1.00	1.00	1.00	1.00	1.00	1.00	1.00	1.00	1.00	1.00	1.00	1.00	1.00	1.00	1.00	1.00	1.00	1.00	1.00	1.00	1.00
**Q64**	1.00	1.00	1.00	1.00	1.00	1.00	1.00	1.00	1.00	1.00	1.00	1.00	1.00	1.00	1.00	1.00	1.00	1.00	1.00	1.00	1.00	1.00	1.00	1.00	1.00	1.00	1.00
**Q65**	1.00	1.00	1.00	1.00	1.00	1.00	1.00	1.00	1.00	1.00	1.00	1.00	1.00	1.00	1.00	1.00	1.00	1.00	1.00	1.00	1.00	1.00	1.00	1.00	1.00	1.00	1.00
**Q66**	1.00	1.00	1.00	1.00	1.00	1.00	1.00	1.00	1.00	1.00	1.00	1.00	1.00	1.00	1.00	1.00	1.00	1.00	1.00	1.00	1.00	1.00	1.00	1.00	1.00	1.00	1.00
**Q67**	1.00	1.00	1.00	1.00	1.00	1.00	1.00	1.00	1.00	1.00	1.00	1.00	1.00	1.00	1.00	1.00	1.00	1.00	1.00	1.00	1.00	1.00	1.00	1.00	1.00	1.00	1.00
**Q68**	1.00	1.00	1.00	1.00	1.00	1.00	1.00	1.00	1.00	1.00	1.00	1.00	1.00	1.00	1.00	1.00	1.00	1.00	1.00	1.00	1.00	1.00	1.00	1.00	1.00	1.00	1.00
**Q69**	0.82	0.64	0.91	0.82	0.64	0.91	0.82	0.64	0.91	0.82	0.64	0.91	0.82	0.64	0.91	0.82	0.64	0.91	0.82	0.64	0.91	0.82	0.64	0.91	0.82	0.64	0.91
**Q70**	0.82	0.64	0.91	0.82	0.64	0.91	0.82	0.64	0.91	0.82	0.64	0.91	0.82	0.64	0.91	0.82	0.64	0.91	0.82	0.64	0.91	0.82	0.64	0.91	0.82	0.64	0.91
**Q71**	1.00	1.00	1.00	1.00	1.00	1.00	1.00	1.00	1.00	1.00	1.00	1.00	1.00	1.00	1.00	1.00	1.00	1.00	1.00	1.00	1.00	1.00	1.00	1.00	1.00	1.00	1.00

Source: Survey 2023.

^a^Q = instrument item

^b^CI = Committee agreement index

^c^kappa = Kappa index

^d^CVI = Content Validity Index.

Particularly about the agreement index between experts (CI), we have all items greater than or equal to 0.80. Using the Kappa index, which assesses the degree of agreement between evaluators on the items under study, we have the following items below 0.80: Behavioral (8, 35, 69 and 70); Objectivity (8, 35, 69 and 70); Simplicity (8, 35, 69 and 70); Clarity (8, 35, 69 and 70); Relevance (35, 69 and 70); Accuracy (8, 35, 69 and 70); Variety (5, 33, 35, 69 and 70); Modality (35, 69 and 70) and Credibility (8, 35, 69 and 70).

Furthermore, through the CVI, defining a cutoff point of 0.80, we have all items under analysis classified as adequate. Therefore, the instrument is being validated by experts.

#### 3. Statistical analysis Module 3

Regarding Module 3, developed for the evaluation of managers related to the ACCRO service, the items that were not contained in Module 2 were evaluated by the group of experts, corresponding to nine items, as shown in Table 4.

**Table 4:** Validation of the instrument for assessing the quality of reception services with obstetric risk classification for managers. Module 3.

**Item**	**Behavioral**			**Objectivity**			**Simplicity**			**Clarity**			**Relevance**			**Precision**			**Variety**			**Modality**			**Credibility**		
**Q** ^**a**^	**CI** ^**b**^	**kappa** ^**c**^	**CVI** ^**d**^	**CI**	**Kappa**	**CVI**	**CI**	**Kappa**	**CVI**	**CI**	**Kappa**	**CVI**	**CI**	**Kappa**	**CVI**	**CI**	**Kappa**	**CVI**	**CI**	**Kappa**	**CVI**	**CI**	**Kappa**	**CVI**	**CI**	**Kappa**	**CVI**
**Q16**	0.80	0.60	0.90	0.78	0.56	0.89	0.80	0.60	0.90	0.80	0.60	0.90	0.80	0.60	0.90	0.80	0.60	0.90	0.80	0.60	0.90	0.80	0.60	0.90	0.80	0.60	0.90
**Q21**	1.00	1.00	1.00	1.00	1.00	1.00	1.00	1.00	1.00	1.00	1.00	1.00	1.00	1.00	1.00	1.00	1.00	1.00	1.00	1.00	1.00	1.00	1.00	1.00	0.80	0.60	0.90
**Q33**	1.00	1.00	1.00	1.00	1.00	1.00	1.00	1.00	1.00	1.00	1.00	1.00	1.00	1.00	1.00	1.00	1.00	1.00	1.00	1.00	1.00	1.00	1.00	1.00	1.00	1.00	1.00
**Q35**	0.80	0.60	0.90	0.80	0.60	0.90	0.80	0.60	0.90	0.80	0.60	0.90	0.80	0.60	0.90	0.80	0.60	0.90	1.00	1.00	1.00	1.00	1.00	1.00	1.00	1.00	1.00
**Q37**	0.80	0.60	0.90	0.80	0.60	0.90	0.80	0.60	0.90	0.80	0.60	0.90	0.80	0.60	0.90	0.80	0.60	0.90	0.80	0.60	0.90	0.80	0.60	0.90	0.80	0.60	0.90
**Q61**	0.80	0.60	0.90	0.80	0.60	0.90	0.80	0.60	0.90	0.80	0.60	0.90	0.80	0.60	0.90	0.80	0.60	0.90	0.80	0.60	0.90	0.80	0.60	0.90	0.80	0.60	0.90
**Q64**	0.80	0.60	0.90	0.80	0.60	0.90	0.80	0.60	0.90	0.80	0.60	0.90	0.80	0.60	0.90	0.80	0.60	0.90	0.80	0.60	0.90	0.80	0.60	0.90	0.80	0.60	0.90
**Q65**	0.80	0.60	0.90	0.80	0.60	0.90	0.80	0.60	0.90	0.80	0.60	0.90	0.80	0.60	0.90	0.80	0.60	0.90	0.64	0.29	0.80	0.80	0.60	0.90	0.80	0.60	0.90
**Q66**	0.80	0.60	0.90	0.80	0.60	0.90	0.80	0.60	0.90	0.80	0.60	0.90	0.80	0.60	0.90	0.80	0.60	0.90	0.64	0.29	0.80	0.80	0.60	0.90	0.80	0.60	0.90

Source: Survey 2023

^a^Q = instrument item

^b^CI = Committee agreement index

^c^kappa = Kappa index

^d^CVI = Content Validity Index

As with the previous modules, the cutoff point for CI, CVI, and Kappa was defined as values equal to or greater than 0.80. Instrument items that obtained values lower than this were excluded from the study.

Namely, regarding the agreement index (CI) between evaluators on the items under study, we have the following items below 0.80: Objectivity (16) and Variety (65 and 66). Using the Kappa index, which assesses the degree of agreement between evaluators on the items under study, we have the following items below 0.80: Behavioral (16, 35, 37, 61, 64, 65 and 66); Objectivity (16, 35, 37, 61, 64, 65 and 66); Simplicity (16, 35, 37, 61, 64, 65 and 66); Clarity (16, 35, 37, 61, 64, 65 and 66); Relevance (16, 35, 37, 61, 64, 65 and 66); Accuracy (16, 35, 37, 61, 64, 65 and 66); Variety (16, 37, 61, 64, 65 and 66); Modality (16, 37, 61, 64, 65 and 66) and Credibility (16, 21, 37, 61, 64, 65 and 66).

Furthermore, through the CVI, defining a cutoff point of 0.80, we have all items under analysis classified as adequate. Therefore, the instrument is being validated with managers, as can be seen in Table 4.

Regarding acceptability, 5 respondents suggested improvements in the wording of the questionnaire and inclusion of other items, with 2 experts also suggesting the exclusion of items because they believed the item was already related to others presented.

Module 1 deals with information relating to access to the service; meeting demand and risk classification; comprehensive care for the user’s health; bonding with the team (effective communication); accountability and coordination of care; assistance received in the sector; respect and preservation of women’s dignity; emotional support; essential physical resources available (structure/ambiance); competent and motivated human resources; satisfaction with the service received during their stay at the service.

Modules 2 and 3, considering the particularities of the participants about the service they offer (qualified health workforce and health management) were made up of elements that provide information regarding accessibility; meeting demand, and risk classification (use of the specific protocol, estimated waiting time, among others); routine care and treatments offered to users; respect and preservation of women’s dignity; emotional support offered to the user; essential physical resources available (structure/ambiance); effective communication with users (and companions) and between team members, as well as with other professionals; intersectionality; information technologies; patient transport and its regulation; competent and motivated human resources; continuing training; team planning and management meetings; participative management; satisfaction regarding the service.

## Discussion

Having the perception of the actors involved in the assistance process in ACCRO services is essential to improve several aspects of the quality of care [22]. This article presents the results of the development and validation of a quality assessment tool for this type of service based on the theoretical framework proposed by Donabedian [9] and the objectives proposed by the WHO [3] and the MS through the Humanization Program in Prenatal and Nascimento [5, 6] for the implementation of ACCRO services, considering the perspective of users, health professionals and managers of these services. To date, no other similar validated tool has been identified that seeks to evaluate these three elements of the care process in a single instrument, which turns out to be a strong point of the study.

The process of developing and validating this questionnaire was based on the guidelines followed in line with the methodology for constructing and validating instruments proposed by Pasquali [17] and had several strengths. The characteristics of the questionnaire were defined in advance, based on prior theoretical knowledge from the scoping study carried out. Expert health professionals with experience participated in this stage of the study as participants in the group of judges, in addition to teachers in the area in question, who were involved in the development process of this phase, including the assessment of content validity, apparent validity, interreliability and observers and acceptability.

The number of items generated for each module (generated seeking to cover all 31 quality statements according to the WHO [3, 14]) can be seen as a limitation. However, this instrument must be considered as a way of evaluating the quality of care in different aspects, which ends up justifying its size.

However, it is understood that when developing questionnaires, the length of the tool must be taken into consideration, so as not to diminish acceptability and ensure feasibility [23–25]. A strategy to ensure that it becomes accepted and viable by the population to be applied, in the next stage (pilot test), is that it is offered in digital format in the three modules and that module 1 is applied in an interview format for users who need to stay longer in the sector due to clinical observation status or who need to remain hospitalized for clinical treatment, as long as they can answer the instrument.

The results suggest that the tool has good content validity. It is worth noting that this is the first stage of the validation process that corresponds to the Theoretical Pole and the beginning of Pasquali’s Empirical Pole [17, 19]. Therefore, other results will be reported separately in future publications.

Its strengths are that the ultimate interest of the tool described in this document is to collaborate with the assessment of the quality of ACCRO services considering the Brazilian health context, but that it can also serve as a guide to identify limitations and edges that interfere in the quality of the health service, to cause changes in routines and conduct, both in the services and in the routines of users.

Furthermore, it is the first quality assessment instrument for this type of care service, which seeks to involve all actors involved in the care process, which is already validated in terms of content and capable of continuing with the following stages of the process. of instrumental validation.

A limitation observed in the study concerns the fact that the instrument contains many items. This question is justified by the fact that such a tool is intended to evaluate different elements in the care process, as well as to observe different aspects of this care.

Another point that lists the limitations of this study is related to the time needed to reach the necessary sample size for all modules (especially modules 2 and 3), considering the population, within the context in which the study was determined. It will be necessary to request authorizations to access other services, as well as respect all project adjustment procedures related to the ethical issues involved.

It is worth mentioning that, for the analysis of data from an instrument to be validated, statistical techniques are required that make important demands, such as factor analysis, so that they can allow the values to produce sufficient variance for the analysis to be consistent, which implies a considerable sample size.

For this reason, the next stages of the study are in the reorganization phase regarding satisfactory access to the sample so that it can continue and complete the validation process, with the completion of the Empirical Pole, executing the Pilot Test, and analytical, with the statistical analyzes that will lead to the conclusion of the psychometric validation.

## Conclusion

The results suggest that the tool has good content validity. It is worth noting that this is the first stage of the validation process that corresponds to the Theoretical Pole and the beginning of Pasquali’s Empirical Pole. As previously stated, the study will continue to conclude the Empirical and Analytical Poles, so that other results will be reported separately in future publications.

## Supporting information

S1 Appendix Guidance on completing the instrument evaluation process by judges/experts. (DOCX)

S2 Appendix Instrument for assessing the quality of care services with obstetric risk classification—Module 1 for users; Module 2 for professionals assisting and Module 3 for service managers. (DOCX)
