# Supplementary material for: Preparation and validation of the content of an instrument to assess the quality of services of reception with risk classification in obstetrics
Source: PLoS One. 2024 Dec 30;19(12):e0315816. doi: 10.1371/journal.pone.0315816 (PMC11684653; doi:10.1371/journal.pone.0315816)
Supplement: S1 Appendix — (DOCX) [file pone.0315816.s001.docx]

**S1 Appendix. Guidance on completing the instrument evaluation process by judges/experts.**

**Dear Judge/Expert,**

Thank you once again for your willingness to participate in our research. This relates ***to the first stage of the study*** in which a group of specialists in the field of Obstetrics will evaluate the items that make up the assessment tool called “Instrument for Assessing the Quality of Reception with Obstetric Risk Classification” (gateway, access to maternal and child services).

To properly fill out the items that make up the tool, it is necessary to follow some guidelines:

1. It is a tool divided into three modules that constitute an instrument for evaluating the quality of Reception with Obstetric Risk Classification, each one aimed at a participant in the care process:
   1. Tool aimed at the User, called “Instrument for assessing the quality of Reception services with Obstetric Risk Classification for **Users - Module 1** ”;
   2. Tool aimed at professionals who assist in the sector (doctors, nurses, and nursing technicians), called “Instrument for assessing the quality of Reception services with Obstetric Risk Classification for **Professionals - Module 2”** ;
   3. Tool intended for Managers, called “Instrument for assessing the quality of Reception services with Obstetric Risk Classification for **Managers - Module 3** ” ;
   4. As this is a group of experts in the area to improve the instrument, its participants must evaluate the three modules (however, the questions will not be repeated. For this reason, about the professional and manager modules, these will not respect an exact numerical sequence in their items).
   5. As this is a very robust instrument, **we kindly ask that the assessment instrument be completed on a notebook or computer.** It most likely cannot be accessed by a cell phone.
2. Each instrument is evaluated according to the “Judgment of items according to the fundamental criteria for item development, according to Pasquali”;
3. Each Instrument must be responded to individually;
4. If you consider it necessary to include an item, leave it written in the field “Important items to be present in this instrument, but absent” **at the end of each module (we suggest that you write down the item you want to add on a sheet of paper so that at the end of the instrument you can write it, since the instrument has several questions, which may result in forgetting when reading other items)** ;
5. If you consider it necessary to exclude an item, leave it written and justify it in the field “Items that should be excluded from this instrument” **found at the end of each module (we suggest that you write down the item you want to exclude on a sheet so that at the end of the instrument you can write it since the instrument has several questions, which may result in forgetting when reading other items)** ;
6. If you consider that an item requires corrections, specify in the field “Comments or suggestions regarding the evaluation of items” **found at the end of each module (we suggest that you write down on a sheet of paper the item you wish to correct or comment on so that at the end of the instrument you can write it since the instrument has several questions, which may result in forgetting when reading other items)** ;

In **evaluating** the items, criteria will be used, according to Pasquali, which aim to add a sense of quality to each item. To provide greater clarity in your analysis, the definition of each criterion was presented in the table below:

| ***Behavioral*** - expresses a clear and precise action;  ***Objectivity*** *-* allows direct, punctual response;  ***Simplicity*** - expresses a single idea;  ***Clarity*** - Understanding clearly and simply;  ***Relevance*** - must not imply an attribute different from that defined. In other words, the phrase must be consistent with the idea of evaluating the quality of care;  ***Precision*** - items do not get confused;  ***Variety*** - do not repeat the same terms in all items;  ***Modality*** - do not use extreme expressions such as, for example, “excellent”, “miserable”;  ***Credibility* -** the item is described in a way that does not seem ridiculous, unreasonable, or childish. |
| --- |

Continuing in the process of evaluating the items, a psychometric scale will be used that will aim to add numerical values to the items, according to what is observed below:

(1) - Does not include the attribute.

(2) - Unable to contemplate the attribute without review.

(3) - Includes the attribute, but requires minimal change

(4) - Includes the attribute.

Finally, it is important to keep in mind, during the evaluation process, that this instrument will evaluate **a specific sector that constitutes a gateway to the hospital service and, as a result, some circumstances that may occur routinely in other sectors generally It is not routine for ACCRO**. For example, postpartum hemorrhage may be observed somewhat more routinely in the delivery department, operating room, or rooming-in. In the ACCRO sector, this circumstance will generally only occur if the woman has access to hemorrhagic syndrome or postpartum hemorrhage that occurred outside the service.

We thank you in advance for your collaboration and contribution! ***You should have between 30 and 50 minutes to complete the assessment of the entire Instrument (the three modules). We ask that you respond within 15 days so that it is possible to continue with the steps of the instrumental validation process.***

If you have any questions or clarifications, please contact the researchers:
