# Supplementary material for: Preparation and validation of the content of an instrument to assess the quality of services of reception with risk classification in obstetrics
Source: PLoS One. 2024 Dec 30;19(12):e0315816. doi: 10.1371/journal.pone.0315816 (PMC11684653; doi:10.1371/journal.pone.0315816)
Supplement: S2 Appendix — (DOCX) [file pone.0315816.s002.docx]

**S2 Appendix. Instrument for Assessing the Quality of care services with obstetric risk classification - Module 1 for users; Module 2 for Professionals assisting and Module 3 for Service Managers.**

**Module 1 for users**

***EVALUATION PROCESS BY JUDGES*:** JUDGMENT OF ITEMS ACCORDING TO THE FUNDAMENTAL CRITERIA FOR PREPARING ITEMS, ACCORDING TO PASQUALI

**Place the number in the space for each attribute**

**(1) - Does not include the attribute**

**(2) - Unable to contemplate the attribute without review.**

**(3) - Includes the attribute, but requires minimal change.**

**(4) - Includes the attribute**

| **ORDER** | **ITEM** | **ITEMS VALUATION ATTRIBUTES** | | | |
| --- | --- | --- | --- | --- | --- |
| **1** | There are spaces at the reception that facilitate your reception and the companion's stay (waiting room). | ( ) Behavioral | ( ) Clarity | | ( ) Variety |
|  |  | ( ) Objectivity | ( ) Relevance | | ( ) Modality |
|  |  | ( ) Simplicity | ( ) Precision | | ( ) Credibility |
| **two** | The Risk Classification room is close to the team. | ( ) Behavioral | ( ) Clarity | | ( ) Variety |
|  |  | ( ) Objectivity | ( ) Relevance | | ( ) Modality |
|  |  | ( ) Simplicity | ( ) Precision | | ( ) Credibility |
| **3** | The Risk Classification room has direct access to both the waiting room and the interior of the hospital. | ( ) Behavioral | ( ) Clarity | | ( ) Variety |
|  |  | ( ) Objectivity | ( ) Relevance | | ( ) Modality |
|  |  | ( ) Simplicity | ( ) Precision | | ( ) Credibility |
| **4** | The environment in the Waiting Room is comfortable and welcoming for you and your companion. | ( ) Behavioral | ( ) Clarity | | ( ) Variety |
|  |  | ( ) Objectivity | ( ) Relevance | | ( ) Modality |
|  |  | ( ) Simplicity | ( ) Precision | | ( ) Credibility |
| **5** | The Waiting Room has several chairs suitable for sitting. | ( ) Behavioral | ( ) Clarity | | ( ) Variety |
|  |  | ( ) Objectivity | ( ) Relevance | | ( ) Modality |
|  |  | ( ) Simplicity | ( ) Precision | | ( ) Credibility |
| **6** | If necessary, there is a place for observation and evaluation of labor that is comfortable and welcoming for your stay. | ( ) Behavioral | ( ) Clarity | | ( ) Variety |
|  |  | ( ) Objectivity | ( ) Relevance | | ( ) Modality |
|  |  | ( ) Simplicity | ( ) Precision | | ( ) Credibility |
| **7** | Observed the presence of drinking fountains. | ( ) Behavioral | ( ) Clarity | | ( ) Variety |
|  |  | ( ) Objectivity | ( ) Relevance | | ( ) Modality |
|  |  | ( ) Simplicity | ( ) Precision | | ( ) Credibility |
| **8** | Observed the presence of audiovisual equipment (TVs). | ( ) Behavioral | ( ) Clarity | | ( ) Variety |
|  |  | ( ) Objectivity | ( ) Relevance | | ( ) Modality |
|  |  | ( ) Simplicity | ( ) Precision | | ( ) Credibility |
| **9** | At reception, you noticed the existence of low benches, with chairs and no barriers for your care/companion. | ( ) Behavioral | ( ) Clarity | | ( ) Variety |
|  |  | ( ) Objectivity | ( ) Relevance | | ( ) Modality |
|  |  | ( ) Simplicity | ( ) Precision | | ( ) Credibility |
| **10** | Comfortable conditions with brightness control (low light) were provided for you. | ( ) Behavioral | ( ) Clarity | | ( ) Variety |
|  |  | ( ) Objectivity | ( ) Relevance | | ( ) Modality |
|  |  | ( ) Simplicity | ( ) Precision | | ( ) Credibility |
| **11** | You were provided with comfortable conditions with temperature control (air-conditioned room). | ( ) Behavioral | ( ) Clarity | | ( ) Variety |
|  |  | ( ) Objectivity | ( ) Relevance | | ( ) Modality |
|  |  | ( ) Simplicity | ( ) Precision | | ( ) Credibility |
| **12** | You were provided with comfortable conditions with noise control in the environment. | ( ) Behavioral | ( ) Clarity | | ( ) Variety |
|  |  | ( ) Objectivity | ( ) Relevance | | ( ) Modality |
|  |  | ( ) Simplicity | ( ) Precision | | ( ) Credibility |
| **13** | There is an accessible signage and visual communication system that is easy to understand and guide. | ( ) Behavioral | ( ) Clarity | | ( ) Variety |
|  |  | ( ) Objectivity | ( ) Relevance | | ( ) Modality |
|  |  | ( ) Simplicity | ( ) Precision | | ( ) Credibility |
| **14** | There is a public Wi-Fi network available to meet users' needs. | ( ) Behavioral | ( ) Clarity | | ( ) Variety |
|  |  | ( ) Objectivity | ( ) Relevance | | ( ) Modality |
|  |  | ( ) Simplicity | ( ) Precision | | ( ) Credibility |
| **15** | Found a public telephone system to meet users' needs. | ( ) Behavioral | ( ) Clarity | | ( ) Variety |
|  |  | ( ) Objectivity | ( ) Relevance | | ( ) Modality |
|  |  | ( ) Simplicity | ( ) Precision | | ( ) Credibility |
| **16** | Observed supply of piped water and basic sanitation in the sector. | ( ) Behavioral | ( ) Clarity | | ( ) Variety |
|  |  | ( ) Objectivity | ( ) Relevance | | ( ) Modality |
|  |  | ( ) Simplicity | ( ) Precision | | ( ) Credibility |
| **17** | There is a supply of electricity in the sector. | ( ) Behavioral | ( ) Clarity | | ( ) Variety |
|  |  | ( ) Objectivity | ( ) Relevance | | ( ) Modality |
|  |  | ( ) Simplicity | ( ) Precision | | ( ) Credibility |
| **18** | Observed a hand-washing sink at the exam site. | ( ) Behavioral | ( ) Clarity | | ( ) Variety |
|  |  | ( ) Objectivity | ( ) Relevance | | ( ) Modality |
|  |  | ( ) Simplicity | ( ) Precision | | ( ) Credibility |
| **19** | There is a hand-washing sink for preparing medicines. | ( ) Behavioral | ( ) Clarity | | ( ) Variety |
|  |  | ( ) Objectivity | ( ) Relevance | | ( ) Modality |
|  |  | ( ) Simplicity | ( ) Precision | | ( ) Credibility |
| **20** | There is a place (counter) for preparing medicines. | ( ) Behavioral | ( ) Clarity | | ( ) Variety |
|  |  | ( ) Objectivity | ( ) Relevance | | ( ) Modality |
|  |  | ( ) Simplicity | ( ) Precision | | ( ) Credibility |
| **21** | I believe that the sector was planned, organized, and maintained correctly to provide care to pregnant/postpartum women. | ( ) Behavioral | ( ) Clarity | | ( ) Variety |
|  |  | ( ) Objectivity | ( ) Relevance | | ( ) Modality |
|  |  | ( ) Simplicity | ( ) Precision | | ( ) Credibility |
| **22** | When you arrived at the service, you went to reception and received instructions there. | ( ) Behavioral | ( ) Clarity | | ( ) Variety |
|  |  | ( ) Objectivity | ( ) Relevance | | ( ) Modality |
|  |  | ( ) Simplicity | ( ) Precision | | ( ) Credibility |
| **23** | The reception professional filled out the service form correctly and completely with speed and clarity of data. | ( ) Behavioral | ( ) Clarity | | ( ) Variety |
|  |  | ( ) Objectivity | ( ) Relevance | | ( ) Modality |
|  |  | ( ) Simplicity | ( ) Precision | | ( ) Credibility |
| **24** | The reception professional sent you to carry out the Risk Classification with the Nurse. | ( ) Behavioral | ( ) Clarity | | ( ) Variety |
|  |  | ( ) Objectivity | ( ) Relevance | | ( ) Modality |
|  |  | ( ) Simplicity | ( ) Precision | | ( ) Credibility |
| **25** | During your stay in the sector, you were guaranteed the presence of the companion of your choice from the moment you arrived at the service. | ( ) Behavioral | ( ) Clarity | | ( ) Variety |
|  |  | ( ) Objectivity | ( ) Relevance | | ( ) Modality |
|  |  | ( ) Simplicity | ( ) Precision | | ( ) Credibility |
| **26** | The staff greeted you in a welcoming manner, calling you by your name. | ( ) Behavioral | ( ) Clarity | | ( ) Variety |
|  |  | ( ) Objectivity | ( ) Relevance | | ( ) Modality |
|  |  | ( ) Simplicity | ( ) Precision | | ( ) Credibility |
| **27** | You and your companion were welcomed cordially and responsibly. | ( ) Behavioral | ( ) Clarity | ( ) Variety | |
|  |  | ( ) Objectivity | ( ) Relevance | ( ) Modality | |
|  |  | ( ) Simplicity | ( ) Precision | ( ) Credibility | |
| **28** | The professionals checked my blood pressure and temperature. | ( ) Behavioral | ( ) Clarity | ( ) Variety | |
|  |  | ( ) Objectivity | ( ) Relevance | ( ) Modality | |
|  |  | ( ) Simplicity | ( ) Precision | ( ) Credibility | |
| **29** | The Nursing technician/Nurse referred you to a doctor after risk classification. | ( ) Behavioral | ( ) Clarity | ( ) Variety | |
|  |  | ( ) Objectivity | ( ) Relevance | ( ) Modality | |
|  |  | ( ) Simplicity | ( ) Precision | ( ) Credibility | |
| **30** | The doctor/nurse/nursing technician paid attention to what you said (your complaints) while you were receiving care. | ( ) Behavioral | ( ) Clarity | ( ) Variety | |
|  |  | ( ) Objectivity | ( ) Relevance | ( ) Modality | |
|  |  | ( ) Simplicity | ( ) Precision | ( ) Credibility | |
| **31** | You were welcomed by the Nurse and went through the risk classification. | ( ) Behavioral | ( ) Clarity | ( ) Variety | |
|  |  | ( ) Objectivity | ( ) Relevance | ( ) Modality | |
|  |  | ( ) Simplicity | ( ) Precision | ( ) Credibility | |
| **32** | She was evaluated by the doctor after having gone through the risk classification by the nurse. | ( ) Behavioral | ( ) Clarity | ( ) Variety | |
|  |  | ( ) Objectivity | ( ) Relevance | ( ) Modality | |
|  |  | ( ) Simplicity | ( ) Precision | ( ) Credibility | |
| **33** | They explained to you how the risk classification occurs and that depending on your health status, you could wait a certain amount of time to be seen by the doctor. | ( ) Behavioral | ( ) Clarity | ( ) Variety | |
|  |  | ( ) Objectivity | ( ) Relevance | ( ) Modality | |
|  |  | ( ) Simplicity | ( ) Precision | ( ) Credibility | |
| **34** | You waited a long time to be seen by the doctor. | ( ) Behavioral | ( ) Clarity | ( ) Variety | |
|  |  | ( ) Objectivity | ( ) Relevance | ( ) Modality | |
|  |  | ( ) Simplicity | ( ) Precision | ( ) Credibility | |
| ***Questions 35, 36, and 37 concern pregnant and postpartum women who have or have had high blood pressure before or during pregnancy. If this is not your case, go to question 38.*** | | | | | |
| **35** | The professionals asked me if I felt a headache, stomach pain, nausea, vomiting, or high blood pressure, or if I saw dark spots in my field of vision. | ( ) Behavioral | ( ) Clarity | ( ) Variety | |
|  |  | ( ) Objectivity | ( ) Relevance | ( ) Modality | |
|  |  | ( ) Simplicity | ( ) Precision | ( ) Credibility | |
| **36** | The professionals requested laboratory tests. | ( ) Behavioral | ( ) Clarity | ( ) Variety | |
|  |  | ( ) Objectivity | ( ) Relevance | ( ) Modality | |
|  |  | ( ) Simplicity | ( ) Precision | ( ) Credibility | |
| **37** | Professionals administered medications when necessary. | ( ) Behavioral | ( ) Clarity | ( ) Variety | |
|  |  | ( ) Objectivity | ( ) Relevance | ( ) Modality | |
|  |  | ( ) Simplicity | ( ) Precision | ( ) Credibility | |
| ***If you experienced postpartum hemorrhage at home or while traveling to the hospital/health service, answer question 38. If this is not your case, go to question 39*.** | | | | | |
| **38** | I received appropriate care for the bleeding situation upon arriving at the sector. | ( ) Behavioral | ( ) Clarity | ( ) Variety | |
|  |  | ( ) Objectivity | ( ) Relevance | ( ) Modality | |
|  |  | ( ) Simplicity | ( ) Precision | ( ) Credibility | |
| **39** | Necessary precautions were taken for my protection by the team, such as washing hands and using masks, and disposable gloves, among others. | ( ) Behavioral | ( ) Clarity | ( ) Variety | |
|  |  | ( ) Objectivity | ( ) Relevance | ( ) Modality | |
|  |  | ( ) Simplicity | ( ) Precision | ( ) Credibility | |
| **40** | The environment where the physical examination was performed was clean and organized. | ( ) Behavioral | ( ) Clarity | ( ) Variety | |
|  |  | ( ) Objectivity | ( ) Relevance | ( ) Modality | |
|  |  | ( ) Simplicity | ( ) Precision | ( ) Credibility | |
| **41** | I received care that caused me discomfort, and embarrassment. | ( ) Behavioral | ( ) Clarity | ( ) Variety | |
|  |  | ( ) Objectivity | ( ) Relevance | ( ) Modality | |
|  |  | ( ) Simplicity | ( ) Precision | ( ) Credibility | |
| **42** | I was subjected to unnecessary or harmful practices. | ( ) Behavioral | ( ) Clarity | ( ) Variety | |
|  |  | ( ) Objectivity | ( ) Relevance | ( ) Modality | |
|  |  | ( ) Simplicity | ( ) Precision | ( ) Credibility | |
| **43** | My family and I received information about the care provided to me and my health status. | ( ) Behavioral | ( ) Clarity | ( ) Variety | |
|  |  | ( ) Objectivity | ( ) Relevance | ( ) Modality | |
|  |  | ( ) Simplicity | ( ) Precision | ( ) Credibility | |
| **44** | There was good interaction between my family, the team, and me. | ( ) Behavioral | ( ) Clarity | ( ) Variety | |
|  |  | ( ) Objectivity | ( ) Relevance | ( ) Modality | |
|  |  | ( ) Simplicity | ( ) Precision | ( ) Credibility | |
| **45** | I was involved in every decision made regarding my care and treatment while I was in the department. | ( ) Behavioral | ( ) Clarity | ( ) Variety | |
|  |  | ( ) Objectivity | ( ) Relevance | ( ) Modality | |
|  |  | ( ) Simplicity | ( ) Precision | ( ) Credibility | |
| **46** | My family and I felt trust in the team when they brought information to us. | ( ) Behavioral | ( ) Clarity | ( ) Variety | |
|  |  | ( ) Objectivity | ( ) Relevance | ( ) Modality | |
|  |  | ( ) Simplicity | ( ) Precision | ( ) Credibility | |
| **47** | My family and I found the information given by the team clear. | ( ) Behavioral | ( ) Clarity | ( ) Variety | |
|  |  | ( ) Objectivity | ( ) Relevance | ( ) Modality | |
|  |  | ( ) Simplicity | ( ) Precision | ( ) Credibility | |
| **48** | If you have difficulty walking, the stretcher bearer will help you with a wheelchair or other form of transport in the sector. | ( ) Behavioral | ( ) Clarity | ( ) Variety | |
|  |  | ( ) Objectivity | ( ) Relevance | ( ) Modality | |
|  |  | ( ) Simplicity | ( ) Precision | ( ) Credibility | |
| **49** | Your privacy was respected while you were in the Reception sector with Obstetric Risk Classification (no unnecessary exposure of your privacy, your body, or the information provided by you and your family). | ( ) Behavioral | ( ) Clarity | ( ) Variety | |
|  |  | ( ) Objectivity | ( ) Relevance | ( ) Modality | |
|  |  | ( ) Simplicity | ( ) Precision | ( ) Credibility | |
| **50** | While I was in the sector I was subjected to situations considered mistreatment. | ( ) Behavioral | ( ) Clarity | ( ) Variety | |
|  |  | ( ) Objectivity | ( ) Relevance | ( ) Modality | |
|  |  | ( ) Simplicity | ( ) Precision | ( ) Credibility | |
| **51** | During my stay in the sector, all the services I received, as well as the interventions (treatments, exams) were explained to me. | ( ) Behavioral | ( ) Clarity | ( ) Variety | |
|  |  | ( ) Objectivity | ( ) Relevance | ( ) Modality | |
|  |  | ( ) Simplicity | ( ) Precision | ( ) Credibility | |
| **52** | After explanation, I agreed that all interventions would be carried out. | ( ) Behavioral | ( ) Clarity | ( ) Variety | |
|  |  | ( ) Objectivity | ( ) Relevance | ( ) Modality | |
|  |  | ( ) Simplicity | ( ) Precision | ( ) Credibility | |
| **53** | I received emotional support from the team and felt strengthened for labor and delivery. | ( ) Behavioral | ( ) Clarity | ( ) Variety | |
|  |  | ( ) Objectivity | ( ) Relevance | ( ) Modality | |
|  |  | ( ) Simplicity | ( ) Precision | ( ) Credibility | |
| **54** | The team welcomed me and my companion cordially and responsibly. | ( ) Behavioral | ( ) Clarity | ( ) Variety | |
|  |  | ( ) Objectivity | ( ) Relevance | ( ) Modality | |
|  |  | ( ) Simplicity | ( ) Precision | ( ) Credibility | |
| **55** | I consider that the professional team that provided me with assistance in the sector was competent in their actions. | ( ) Behavioral | ( ) Clarity | ( ) Variety | |
|  |  | ( ) Objectivity | ( ) Relevance | ( ) Modality | |
|  |  | ( ) Simplicity | ( ) Precision | ( ) Credibility | |
| **56** | I believe that the sector team was motivated to work. | ( ) Behavioral | ( ) Clarity | ( ) Variety | |
|  |  | ( ) Objectivity | ( ) Relevance | ( ) Modality | |
|  |  | ( ) Simplicity | ( ) Precision | ( ) Credibility | |
| **57** | Workers in the sector team do not have good communication with each other. | ( ) Behavioral | ( ) Clarity | ( ) Variety | |
|  |  | ( ) Objectivity | ( ) Relevance | ( ) Modality | |
|  |  | ( ) Simplicity | ( ) Precision | ( ) Credibility | |
| **58** | The number of professionals is not sufficient to carry out the work. | ( ) Behavioral | ( ) Clarity | ( ) Variety | |
|  |  | ( ) Objectivity | ( ) Relevance | ( ) Modality | |
|  |  | ( ) Simplicity | ( ) Precision | ( ) Credibility | |
| **59** | The sequence of care occurs according to the severity of the case and not according to the order of arrival. | ( ) Behavioral | ( ) Clarity | ( ) Variety | |
|  |  | ( ) Objectivity | ( ) Relevance | ( ) Modality | |
|  |  | ( ) Simplicity | ( ) Precision | ( ) Credibility | |
| **60** | Respect and humanized care are not present in the sector. | ( ) Behavioral | ( ) Clarity | ( ) Variety | |
|  |  | ( ) Objectivity | ( ) Relevance | ( ) Modality | |
|  |  | ( ) Simplicity | ( ) Precision | ( ) Credibility | |
| **61** | Professionals working in the sector work in an integrated manner. | ( ) Behavioral | ( ) Clarity | ( ) Variety | |
|  |  | ( ) Objectivity | ( ) Relevance | ( ) Modality | |
|  |  | ( ) Simplicity | ( ) Precision | ( ) Credibility | |
| **62** | The wait for service is long. It does not respect the waiting time that was communicated to me. | ( ) Behavioral | ( ) Clarity | ( ) Variety | |
|  |  | ( ) Objectivity | ( ) Relevance | ( ) Modality | |
|  |  | ( ) Simplicity | ( ) Precision | ( ) Credibility | |
| **63** | The service does not solve the problem that brought me to the service. | ( ) Behavioral | ( ) Clarity | ( ) Variety | |
|  |  | ( ) Objectivity | ( ) Relevance | ( ) Modality | |
|  |  | ( ) Simplicity | ( ) Precision | ( ) Credibility | |
| **64** | I feel satisfied with the service I received in the sector. | ( ) Behavioral | ( ) Clarity | ( ) Variety | |
|  |  | ( ) Objectivity | ( ) Relevance | ( ) Modality | |
|  |  | ( ) Simplicity | ( ) Precision | ( ) Credibility | |

Items that are important to be present in this instrument, but were not included (highlight the dimension to which this item would be associated - structure, process, or result):

_____________________________________________________________________________________________________________________________________________________________________________________________________________________________________________________________________________________________________________________________________________________

Items that should be excluded from this instrument (please justify the need to exclude the item):

_____________________________________________________________________________________________________________________________________________________________________________________________________________________________________________________________________________________________________________________________________________________

Comments or suggestions regarding the evaluation of the items:

_____________________________________________________________________________________________________________________________________________________________________________________________________________________________________________________________________________________________________________________________________________________

**Module 2 for Professionals providing assistance**

***EVALUATION PROCESS BY JUDGES:* JUDGMENT OF ITEMS ACCORDING TO THE FUNDAMENTAL CRITERIA FOR PREPARING ITEMS, ACCORDING TO PASQUALI**

**Place the number in the space for each attribute**

**(1) - Does not include the attribute**

**(2) - Unable to contemplate the attribute without review.**

**(3) - Includes the attribute, but requires minimal change.**

**(4) - Includes the attribute**

| **ORDER** | **ITEM** | **ITEMS VALUATION ATTRIBUTES** | | | |
| --- | --- | --- | --- | --- | --- |
| **1** | The service routinely assesses and classifies the risk of pregnant and postpartum women in an appropriate and timely manner, considering what is recommended by the WHO and MS. | ( ) Behavioral | ( ) Clarity | | ( ) Variety |
|  |  | ( ) Objectivity | ( ) Relevance | | ( ) Modality |
|  |  | ( ) Simplicity | ( ) Precision | | ( ) Credibility |
| **two** | The service carries out, when necessary, reevaluation of the pregnant/postpartum woman, taking into account signs and symptoms that may present a risk that require reclassification, by what is recommended by the WHO and MS. | ( ) Behavioral | ( ) Clarity | | ( ) Variety |
|  |  | ( ) Objectivity | ( ) Relevance | | ( ) Modality |
|  |  | ( ) Simplicity | ( ) Precision | | ( ) Credibility |
| **3** | The reception professional guides the pregnant/postpartum woman and her family and directs them to the service. | ( ) Behavioral | ( ) Clarity | | ( ) Variety |
|  |  | ( ) Objectivity | ( ) Relevance | | ( ) Modality |
|  |  | ( ) Simplicity | ( ) Precision | | ( ) Credibility |
| **4** | The reception professional correctly fills out the service form with agility and clear data. | ( ) Behavioral | ( ) Clarity | | ( ) Variety |
|  |  | ( ) Objectivity | ( ) Relevance | | ( ) Modality |
|  |  | ( ) Simplicity | ( ) Precision | | ( ) Credibility |
| **5** | The reception professional fills out the service form with speed and clear data. | ( ) Behavioral | ( ) Clarity | | ( ) Variety |
|  |  | ( ) Objectivity | ( ) Relevance | | ( ) Modality |
|  |  | ( ) Simplicity | ( ) Precision | | ( ) Credibility |
| **6** | The reception professional directs the pregnant/postpartum woman to carry out the Risk Classification with the Nurse. | ( ) Behavioral | ( ) Clarity | | ( ) Variety |
|  |  | ( ) Objectivity | ( ) Relevance | | ( ) Modality |
|  |  | ( ) Simplicity | ( ) Precision | | ( ) Credibility |
| **7** | The Nursing technician measures the patients' vital signs. | ( ) Behavioral | ( ) Clarity | | ( ) Variety |
|  |  | ( ) Objectivity | ( ) Relevance | | ( ) Modality |
|  |  | ( ) Simplicity | ( ) Precision | | ( ) Credibility |
| **8** | The doctor/nurse/nursing technician pays attention to the complaints brought by pregnant/postpartum women, through active listening. | ( ) Behavioral | ( ) Clarity | | ( ) Variety |
|  |  | ( ) Objectivity | ( ) Relevance | | ( ) Modality |
|  |  | ( ) Simplicity | ( ) Precision | | ( ) Credibility |
| **9** | All pregnant/postpartum women are welcomed by the Nurse and undergo risk classification. | ( ) Behavioral | ( ) Clarity | | ( ) Variety |
|  |  | ( ) Objectivity | ( ) Relevance | | ( ) Modality |
|  |  | ( ) Simplicity | ( ) Precision | | ( ) Credibility |
| **10** | Pregnant/postpartum women are evaluated by the doctor after having gone through the risk classification by the nurse. | ( ) Behavioral | ( ) Clarity | | ( ) Variety |
|  |  | ( ) Objectivity | ( ) Relevance | | ( ) Modality |
|  |  | ( ) Simplicity | ( ) Precision | | ( ) Credibility |
| **11** | It is routine for the service to explain how the risk classification occurs and the waiting time to be seen by the doctor. | ( ) Behavioral | ( ) Clarity | | ( ) Variety |
|  |  | ( ) Objectivity | ( ) Relevance | | ( ) Modality |
|  |  | ( ) Simplicity | ( ) Precision | | ( ) Credibility |
| **12** | All women receive care by standard precautions to prevent hospital-acquired infections, such as hand washing and, the use of disposable gloves, and masks. | ( ) Behavioral | ( ) Clarity | | ( ) Variety |
|  |  | ( ) Objectivity | ( ) Relevance | | ( ) Modality |
|  |  | ( ) Simplicity | ( ) Precision | | ( ) Credibility |
| **13** | The environment where the physical examination is carried out is always clean and organized. | ( ) Behavioral | ( ) Clarity | | ( ) Variety |
|  |  | ( ) Objectivity | ( ) Relevance | | ( ) Modality |
|  |  | ( ) Simplicity | ( ) Precision | | ( ) Credibility |
| **14** | The patient's privacy is respected while in the Reception sector with Obstetric Risk Classification (no unnecessary exposure of her intimacy, her body, or the information provided by her and her family). | ( ) Behavioral | ( ) Clarity | | ( ) Variety |
|  |  | ( ) Objectivity | ( ) Relevance | | ( ) Modality |
|  |  | ( ) Simplicity | ( ) Precision | | ( ) Credibility |
| **15** | During the patient's stay in the sector, all the services they receive, as well as the interventions (treatments, exams) are explained to them. | ( ) Behavioral | ( ) Clarity | | ( ) Variety |
|  |  | ( ) Objectivity | ( ) Relevance | | ( ) Modality |
|  |  | ( ) Simplicity | ( ) Precision | | ( ) Credibility |
| **16** | All interventions are carried out after informed consent from the patient. | ( ) Behavioral | ( ) Clarity | | ( ) Variety |
|  |  | ( ) Objectivity | ( ) Relevance | | ( ) Modality |
|  |  | ( ) Simplicity | ( ) Precision | | ( ) Credibility |
| **17** | The sector team is motivated to work. | ( ) Behavioral | ( ) Clarity | | ( ) Variety |
|  |  | ( ) Objectivity | ( ) Relevance | | ( ) Modality |
|  |  | ( ) Simplicity | ( ) Precision | | ( ) Credibility |
| **18** | As a professional in the sector, I feel motivated to work. | ( ) Behavioral | ( ) Clarity | | ( ) Variety |
|  |  | ( ) Objectivity | ( ) Relevance | | ( ) Modality |
|  |  | ( ) Simplicity | ( ) Precision | | ( ) Credibility |
| **19** | The sector team workers have good communication with each other. | ( ) Behavioral | ( ) Clarity | | ( ) Variety |
|  |  | ( ) Objectivity | ( ) Relevance | | ( ) Modality |
|  |  | ( ) Simplicity | ( ) Precision | | ( ) Credibility |
| **20** | The number of professionals is sufficient to carry out the work. | ( ) Behavioral | ( ) Clarity | | ( ) Variety |
|  |  | ( ) Objectivity | ( ) Relevance | | ( ) Modality |
|  |  | ( ) Simplicity | ( ) Precision | | ( ) Credibility |
| **22** | There is support for continuous improvement of the quality of service in the sector by the sector/service management. | ( ) Behavioral | ( ) Clarity | | ( ) Variety |
|  |  | ( ) Objectivity | ( ) Relevance | | ( ) Modality |
|  |  | ( ) Simplicity | ( ) Precision | | ( ) Credibility |
| **23** | You feel adequately qualified to perform your role. | ( ) Behavioral | ( ) Clarity | | ( ) Variety |
|  |  | ( ) Objectivity | ( ) Relevance | | ( ) Modality |
|  |  | ( ) Simplicity | ( ) Precision | | ( ) Credibility |
| ***Questions 24, 25, and 26 refer to patients who arrived at the service with a clinical condition related to hypertensive syndromes.*** | | | | | |
| **24** | Women with pre-eclampsia/eclampsia who arrive at the service/sector promptly receive appropriate interventions by WHO and MS recommendations. | ( ) Behavioral | ( ) Clarity | | ( ) Variety |
|  |  | ( ) Objectivity | ( ) Relevance | | ( ) Modality |
|  |  | ( ) Simplicity | ( ) Precision | | ( ) Credibility |
| **25** | The laboratory service can provide the results of laboratory tests necessary for a differential diagnosis of mild and severe pre-eclampsia promptly. | ( ) Behavioral | ( ) Clarity | | ( ) Variety |
|  |  | ( ) Objectivity | ( ) Relevance | | ( ) Modality |
|  |  | ( ) Simplicity | ( ) Precision | | ( ) Credibility |
| **26** | Your institution's laboratory collaborates quickly with the Reception with Risk Classification sector, working in an interconnected and interprofessional way. | ( ) Behavioral | ( ) Clarity | | ( ) Variety |
|  |  | ( ) Objectivity | ( ) Relevance | | ( ) Modality |
|  |  | ( ) Simplicity | ( ) Precision | | ( ) Credibility |
| **27** | Women who arrive at the sector with hemorrhagic syndrome or postpartum hemorrhage after a home birth or during the journey to the hospital, promptly receive appropriate interventions, by what is recommended by the WHO and MS. | ( ) Behavioral | ( ) Clarity | ( ) Variety | |
|  |  | ( ) Objectivity | ( ) Relevance | ( ) Modality | |
|  |  | ( ) Simplicity | ( ) Precision | ( ) Credibility | |
| **28** | Women who arrive at the service with suggestive symptoms and/or laboratory test results compatible with an infectious condition promptly receive appropriate interventions, by WHO and MOH guidelines. | ( ) Behavioral | ( ) Clarity | ( ) Variety | |
|  |  | ( ) Objectivity | ( ) Relevance | ( ) Modality | |
|  |  | ( ) Simplicity | ( ) Precision | ( ) Credibility | |
| **29** | In its service, no woman is subjected to unnecessary or harmful practices. | ( ) Behavioral | ( ) Clarity | ( ) Variety | |
|  |  | ( ) Objectivity | ( ) Relevance | ( ) Modality | |
|  |  | ( ) Simplicity | ( ) Precision | ( ) Credibility | |
| **30** | In its service, no woman experiences circumstances that cause discomfort or embarrassment. | ( ) Behavioral | ( ) Clarity | ( ) Variety | |
|  |  | ( ) Objectivity | ( ) Relevance | ( ) Modality | |
|  |  | ( ) Simplicity | ( ) Precision | ( ) Credibility | |
| **31** | In the sector, every woman has a complete, accurate medical record that follows a standard. | ( ) Behavioral | ( ) Clarity | ( ) Variety | |
|  |  | ( ) Objectivity | ( ) Relevance | ( ) Modality | |
|  |  | ( ) Simplicity | ( ) Precision | ( ) Credibility | |
| **32** | Training is offered to collect data using the standardized form. | ( ) Behavioral | ( ) Clarity | ( ) Variety | |
|  |  | ( ) Objectivity | ( ) Relevance | ( ) Modality | |
|  |  | ( ) Simplicity | ( ) Precision | ( ) Credibility | |
| **33** | The data collected by the form is periodically analyzed and feedback is provided to the team as a way to monitor and improve their performance. | ( ) Behavioral | ( ) Clarity | ( ) Variety | |
|  |  | ( ) Objectivity | ( ) Relevance | ( ) Modality | |
|  |  | ( ) Simplicity | ( ) Precision | ( ) Credibility | |
| **34** | In the sector, all women are adequately evaluated, so that they can be referred to other sectors or services quickly. | ( ) Behavioral | ( ) Clarity | ( ) Variety | |
|  |  | ( ) Objectivity | ( ) Relevance | ( ) Modality | |
|  |  | ( ) Simplicity | ( ) Precision | ( ) Credibility | |
| **35** | The Regulatory Service of your municipality/state or communication between services, when referral is necessary, works properly and without delay. | ( ) Behavioral | ( ) Clarity | ( ) Variety | |
|  |  | ( ) Objectivity | ( ) Relevance | ( ) Modality | |
|  |  | ( ) Simplicity | ( ) Precision | ( ) Credibility | |
| **36** | For each woman referred within the service to other sectors or other health services, there is an exchange of appropriate information and feedback regarding the patient's clinical condition to your team when you refer them. | ( ) Behavioral | ( ) Clarity | ( ) Variety | |
|  |  | ( ) Objectivity | ( ) Relevance | ( ) Modality | |
|  |  | ( ) Simplicity | ( ) Precision | ( ) Credibility | |
| **37** | When you receive a patient referred from another service, information is exchanged, with you providing feedback to the team that referred the patient. | ( ) Behavioral | ( ) Clarity | ( ) Variety | |
|  |  | ( ) Objectivity | ( ) Relevance | ( ) Modality | |
|  |  | ( ) Simplicity | ( ) Precision | ( ) Credibility | |
| **38** | Within your team, communication occurs effectively between professionals. | ( ) Behavioral | ( ) Clarity | ( ) Variety | |
|  |  | ( ) Objectivity | ( ) Relevance | ( ) Modality | |
|  |  | ( ) Simplicity | ( ) Precision | ( ) Credibility | |
| **39** | You, as a professional, can maintain effective communication with the patient and her family, offering clear information about her care and treatment. | ( ) Behavioral | ( ) Clarity | ( ) Variety | |
|  |  | ( ) Objectivity | ( ) Relevance | ( ) Modality | |
|  |  | ( ) Simplicity | ( ) Precision | ( ) Credibility | |
| **40** | The service respects and guarantees pregnant/postpartum women the right to the companion of their choice, by Federal Law No. 11,108/2005 (Companion Law). | ( ) Behavioral | ( ) Clarity | ( ) Variety | |
|  |  | ( ) Objectivity | ( ) Relevance | ( ) Modality | |
|  |  | ( ) Simplicity | ( ) Precision | ( ) Credibility | |
| **41** | The team strives to emotionally support the pregnant woman who arrives at the sector so that she can feel strengthened in her capacity for the birth process. | ( ) Behavioral | ( ) Clarity | ( ) Variety | |
|  |  | ( ) Objectivity | ( ) Relevance | ( ) Modality | |
|  |  | ( ) Simplicity | ( ) Precision | ( ) Credibility | |
| **42** | The team strives to emotionally support the pregnant woman who arrives at the sector so that she can feel welcomed during the birth process. | ( ) Behavioral | ( ) Clarity | ( ) Variety | |
|  |  | ( ) Objectivity | ( ) Relevance | ( ) Modality | |
|  |  | ( ) Simplicity | ( ) Precision | ( ) Credibility | |
| **43** | There is at least one well-trained and competent qualified midwife or obstetrician (doctor or nurse) on your team, present 24 hours a day in the sector. | ( ) Behavioral | ( ) Clarity | ( ) Variety | |
|  |  | ( ) Objectivity | ( ) Relevance | ( ) Modality | |
|  |  | ( ) Simplicity | ( ) Precision | ( ) Credibility | |
| **44** | The sector's team has the appropriate competence and combination of skills to serve the sector, considering the specificities of pregnancy, childbirth, and the postpartum period. | ( ) Behavioral | ( ) Clarity | ( ) Variety | |
|  |  | ( ) Objectivity | ( ) Relevance | ( ) Modality | |
|  |  | ( ) Simplicity | ( ) Precision | ( ) Credibility | |
| **45** | There are spaces at the reception that facilitate the reception and stay of the companion (waiting room) | ( ) Behavioral | ( ) Clarity | ( ) Variety | |
|  |  | ( ) Objectivity | ( ) Relevance | ( ) Modality | |
|  |  | ( ) Simplicity | ( ) Precision | ( ) Credibility | |
| **46** | There is a Risk Classification room close to the reception team, with direct access to both the waiting room and the interior of the hospital. | ( ) Behavioral | ( ) Clarity | ( ) Variety | |
|  |  | ( ) Objectivity | ( ) Relevance | ( ) Modality | |
|  |  | ( ) Simplicity | ( ) Precision | ( ) Credibility | |
| **47** | The environment in the Waiting Room is comfortable for pregnant/postpartum women and their companions, with a welcoming space and an adequate number of chairs. | ( ) Behavioral | ( ) Clarity | ( ) Variety | |
|  |  | ( ) Objectivity | ( ) Relevance | ( ) Modality | |
|  |  | ( ) Simplicity | ( ) Precision | ( ) Credibility | |
| **48** | If necessary, there is a place for observation and evaluation of labor that is comfortable and welcoming for the pregnant woman to stay. | ( ) Behavioral | ( ) Clarity | ( ) Variety | |
|  |  | ( ) Objectivity | ( ) Relevance | ( ) Modality | |
|  |  | ( ) Simplicity | ( ) Precision | ( ) Credibility | |
| **49** | Drinking fountains and audiovisual equipment (TVs) are available. | ( ) Behavioral | ( ) Clarity | ( ) Variety | |
|  |  | ( ) Objectivity | ( ) Relevance | ( ) Modality | |
|  |  | ( ) Simplicity | ( ) Precision | ( ) Credibility | |
| **50** | At reception, there are low benches, with chairs and no barriers to assist pregnant women and their companions. | ( ) Behavioral | ( ) Clarity | ( ) Variety | |
|  |  | ( ) Objectivity | ( ) Relevance | ( ) Modality | |
|  |  | ( ) Simplicity | ( ) Precision | ( ) Credibility | |
| **51** | There are conditions to improve comfort with brightness control (low light). | ( ) Behavioral | ( ) Clarity | ( ) Variety | |
|  |  | ( ) Objectivity | ( ) Relevance | ( ) Modality | |
|  |  | ( ) Simplicity | ( ) Precision | ( ) Credibility | |
| **52** | There are conditions to improve comfort with temperature control (air-conditioned room). | ( ) Behavioral | ( ) Clarity | ( ) Variety | |
|  |  | ( ) Objectivity | ( ) Relevance | ( ) Modality | |
|  |  | ( ) Simplicity | ( ) Precision | ( ) Credibility | |
| **53** | There are conditions to improve comfort with noise control in the environment. | ( ) Behavioral | ( ) Clarity | ( ) Variety | |
|  |  | ( ) Objectivity | ( ) Relevance | ( ) Modality | |
|  |  | ( ) Simplicity | ( ) Precision | ( ) Credibility | |
| **56** | There is a public telephone system to meet users' needs. | ( ) Behavioral | ( ) Clarity | ( ) Variety | |
|  |  | ( ) Objectivity | ( ) Relevance | ( ) Modality | |
|  |  | ( ) Simplicity | ( ) Precision | ( ) Credibility | |
| **57** | There is a supply of piped water, electricity, and basic sanitation in the sector. | ( ) Behavioral | ( ) Clarity | ( ) Variety | |
|  |  | ( ) Objectivity | ( ) Relevance | ( ) Modality | |
|  |  | ( ) Simplicity | ( ) Precision | ( ) Credibility | |
| **58** | There is a hand-washing sink on site for exams and medication preparation. | ( ) Behavioral | ( ) Clarity | ( ) Variety | |
|  |  | ( ) Objectivity | ( ) Relevance | ( ) Modality | |
|  |  | ( ) Simplicity | ( ) Precision | ( ) Credibility | |
| **60** | The Obstetric Risk Classification Reception sector area is designed, organized, and maintained so that each woman can be attended to according to her particular needs, facilitating continuity of care. | ( ) Behavioral | ( ) Clarity | ( ) Variety | |
|  |  | ( ) Objectivity | ( ) Relevance | ( ) Modality | |
|  |  | ( ) Simplicity | ( ) Precision | ( ) Credibility | |
| **61** | Regarding the patient's Risk Classification, the service provides a standardized record that follows the Obstetric Risk protocols directed by the Ministry of Health for Risk Classification in pregnant and postpartum women. | ( ) Behavioral | ( ) Clarity | ( ) Variety | |
|  |  | ( ) Objectivity | ( ) Relevance | ( ) Modality | |
|  |  | ( ) Simplicity | ( ) Precision | ( ) Credibility | |
| **62** | There is a standard to differentiate the patient according to the risk classification received (for example: use of colors in medical records, bracelets indicating colors). | ( ) Behavioral | ( ) Clarity | ( ) Variety | |
|  |  | ( ) Objectivity | ( ) Relevance | ( ) Modality | |
|  |  | ( ) Simplicity | ( ) Precision | ( ) Credibility | |
| **63** | Standardized patient registration forms are digitized. | ( ) Behavioral | ( ) Clarity | ( ) Variety | |
|  |  | ( ) Objectivity | ( ) Relevance | ( ) Modality | |
|  |  | ( ) Simplicity | ( ) Precision | ( ) Credibility | |
| **64** | Standardized patient registration forms are available online. | ( ) Behavioral | ( ) Clarity | ( ) Variety | |
|  |  | ( ) Objectivity | ( ) Relevance | ( ) Modality | |
|  |  | ( ) Simplicity | ( ) Precision | ( ) Credibility | |
| **65** | There is an adequate place for hand washing and waste disposal that can meet the needs of employees, women, and their families. | ( ) Behavioral | ( ) Clarity | ( ) Variety | |
|  |  | ( ) Objectivity | ( ) Relevance | ( ) Modality | |
|  |  | ( ) Simplicity | ( ) Precision | ( ) Credibility | |
| **66** | The service provides supplies, equipment, and medications as needed and in sufficient quantities to provide timely assistance for routine care and treatment of complications. | ( ) Behavioral | ( ) Clarity | ( ) Variety | |
|  |  | ( ) Objectivity | ( ) Relevance | ( ) Modality | |
|  |  | ( ) Simplicity | ( ) Precision | ( ) Credibility | |
| **67** | Respect and humanized care are present in the sector. | ( ) Behavioral | ( ) Clarity | ( ) Variety | |
|  |  | ( ) Objectivity | ( ) Relevance | ( ) Modality | |
|  |  | ( ) Simplicity | ( ) Precision | ( ) Credibility | |
| **68** | The professionals who work in the ACCRO sector work in an integrated manner with each other. | ( ) Behavioral | ( ) Clarity | ( ) Variety | |
|  |  | ( ) Objectivity | ( ) Relevance | ( ) Modality | |
|  |  | ( ) Simplicity | ( ) Precision | ( ) Credibility | |
| **69** | The wait for service is long. Does not respect the waiting time as recommended by the Ministry of Health for each color/risk classification category. | ( ) Behavioral | ( ) Clarity | ( ) Variety | |
|  |  | ( ) Objectivity | ( ) Relevance | ( ) Modality | |
|  |  | ( ) Simplicity | ( ) Precision | ( ) Credibility | |
| **70** | The service is not resolutive. | ( ) Behavioral | ( ) Clarity | ( ) Variety | |
|  |  | ( ) Objectivity | ( ) Relevance | ( ) Modality | |
|  |  | ( ) Simplicity | ( ) Precision | ( ) Credibility | |
| **71** | Professionals working in the ACCRO sector are satisfied. | ( ) Behavioral | ( ) Clarity | ( ) Variety | |
|  |  | ( ) Objectivity | ( ) Relevance | ( ) Modality | |
|  |  | ( ) Simplicity | ( ) Precision | ( ) Credibility | |

Items that are important to be present in this instrument, but were not included (highlight the dimension to which this item would be associated - structure, process, or result):

_____________________________________________________________________________________________________________________________________________________________________________________________________________________________________________________________________________________________________________________________________________________

Items that should be excluded from this instrument (please justify the need to exclude the item):

_____________________________________________________________________________________________________________________________________________________________________________________________________________________________________________________________________________________________________________________________________________________

Comments or suggestions regarding the evaluation of the items:

_____________________________________________________________________________________________________________________________________________________________________________________________________________________________________________________________________________________________________________________________________________________

**Module 3 for Service Managers**

***EVALUATION PROCESS BY JUDGES*: JUDGMENT OF ITEMS ACCORDING TO THE FUNDAMENTAL CRITERIA FOR PREPARING ITEMS, ACCORDING TO PASQUALI.**

**Place the number in the space for each attribute**

**(1) - Does not include the attribute**

**(2) - Unable to contemplate the attribute without review.**

**(3) - Includes the attribute, but requires minimal change.**

**(4) - Includes the attribute**

| **ORDER** | **ITEM** | **ITEMS VALUATION ATTRIBUTES** | | | |
| --- | --- | --- | --- | --- | --- |
| **16** | As a service manager, I receive many complaints from the team that indicate a lack of motivation for work. | ( ) Behavioral | ( ) Clarity | | ( ) Variety |
|  |  | ( ) Objectivity | ( ) Relevance | | ( ) Modality |
|  |  | ( ) Simplicity | ( ) Precision | | ( ) Credibility |
| **21** | There is support from management for ongoing education to adequately qualify the team to perform their duties. | ( ) Behavioral | ( ) Clarity | | ( ) Variety |
|  |  | ( ) Objectivity | ( ) Relevance | | ( ) Modality |
|  |  | ( ) Simplicity | ( ) Precision | | ( ) Credibility |
| **33** | Within the service team, communication occurs effectively between professionals. | ( ) Behavioral | ( ) Clarity | ( ) Variety | |
|  |  | ( ) Objectivity | ( ) Relevance | ( ) Modality | |
|  |  | ( ) Simplicity | ( ) Precision | ( ) Credibility | |
| **35** | There is at least one well-trained and competent qualified midwife or obstetrician (doctor or nurse) on your team, present 24 hours a day in the sector. | ( ) Behavioral | ( ) Clarity | ( ) Variety | |
|  |  | ( ) Objectivity | ( ) Relevance | ( ) Modality | |
|  |  | ( ) Simplicity | ( ) Precision | ( ) Credibility | |
| **37** | There is an effort by the management that governs the sector (administrative and clinical) to implement appropriate policies that support continuous improvement in the quality of care. | ( ) Behavioral | ( ) Clarity | ( ) Variety | |
|  |  | ( ) Objectivity | ( ) Relevance | ( ) Modality | |
|  |  | ( ) Simplicity | ( ) Precision | ( ) Credibility | |
| **61** | The professionals who work in the ACCRO sector work in an integrated manner with each other. | ( ) Behavioral | ( ) Clarity | ( ) Variety | |
|  |  | ( ) Objectivity | ( ) Relevance | ( ) Modality | |
|  |  | ( ) Simplicity | ( ) Precision | ( ) Credibility | |
| **64** | Professionals working in the ACCRO sector are satisfied. | ( ) Behavioral | ( ) Clarity | ( ) Variety | |
|  |  | ( ) Objectivity | ( ) Relevance | ( ) Modality | |
|  |  | ( ) Simplicity | ( ) Precision | ( ) Credibility | |
| **65** | Management is concerned with offering permanent education seeking professional qualification | ( ) Behavioral | ( ) Clarity | ( ) Variety | |
|  |  | ( ) Objectivity | ( ) Relevance | ( ) Modality | |
|  |  | ( ) Simplicity | ( ) Precision | ( ) Credibility | |
| **66** | Management plans implements, and continuously evaluates the sector's actions, seeking to improve the quality offered to customers. | ( ) Behavioral | ( ) Clarity | ( ) Variety | |
|  |  | ( ) Objectivity | ( ) Relevance | ( ) Modality | |
|  |  | ( ) Simplicity | ( ) Precision | ( ) Credibility | |

Items that are important to be present in this instrument, but were not included (highlight the dimension to which this item would be associated - structure, process, or result):

_____________________________________________________________________________________________________________________________________________________________________________________________________________________________________________________________________________________________________________________________________________________

Items that should be excluded from this instrument (please justify the need to exclude the item):

_____________________________________________________________________________________________________________________________________________________________________________________________________________________________________________________________________________________________________________________________________________________

Comments or suggestions regarding the evaluation of the items:

_____________________________________________________________________________________________________________________________________________________________________________________________________________________________________________________________________________________________________________________________________________________

**We thank you for your collaboration in the first part of this study.**
